# How people reason with counterfactual and causal explanations for Artificial Intelligence decisions in familiar and unfamiliar domains

**DOI:** 10.3758/s13421-023-01407-5

**Published:** 2023-03-24

**Authors:** Lenart Celar, Ruth M. J. Byrne

**Affiliations:** School of Psychology and Institute of Neuroscience, Trinity College Dublin, University of Dublin, Dublin, Ireland

**Keywords:** Counterfactuals, Causals, Explanations, Decisions, AI decision support systems

## Abstract

Few empirical studies have examined how people understand counterfactual explanations for other people’s decisions, for example, “*if you had asked for a lower amount, your loan application would have been approved”.* Yet many current Artificial Intelligence (AI) decision support systems rely on counterfactual explanations to improve human understanding and trust. We compared counterfactual explanations to causal ones, i.e., *“because you asked for a high amount, your loan application was not approved*”, for an AI’s decisions in a familiar domain (alcohol and driving) and an unfamiliar one (chemical safety) in four experiments (n = 731). Participants were shown inputs to an AI system, its decisions, and an explanation for each decision; they attempted to predict the AI’s decisions, or to make their own decisions. Participants judged counterfactual explanations more helpful than causal ones, but counterfactuals did not improve the accuracy of their predictions of the AI’s decisions more than causals (Experiment 1). However, counterfactuals improved the accuracy of participants’ own decisions more than causals (Experiment 2). When the AI’s decisions were correct (Experiments 1 and 2), participants considered explanations more helpful and made more accurate judgements in the familiar domain than in the unfamiliar one; but when the AI’s decisions were incorrect, they considered explanations less helpful and made fewer accurate judgements in the familiar domain than the unfamiliar one, whether they predicted the AI’s decisions (Experiment 3a) or made their own decisions (Experiment 3b). The results corroborate the proposal that counterfactuals provide richer information than causals, because their mental representation includes more possibilities.

**Supplementary Information:**

The online version contains supplementary material available at 10.3758/s13421-023-01407-5.

## Introduction

Suppose you have had a few glasses of wine over a long dinner at a family gathering and you are wondering whether you are legally over or under the limit to drive home safely. You have downloaded an Artificial Intelligence (AI) decision support system to your smartphone and you decide to use it for the first time. You enter the information it requires: the number of units you have drunk, the duration over which you drank, and whether you have eaten, as well as details such as your weight and gender, and it provides you with a decision: *You are under the limit*. It also provides you with an explanation for its decision: *You would have been over the limit if you had drunk on an empty stomach.* Others at the gathering want to know what it says about them, and soon you are entering details about various cousins, aunts, uncles, and grandparents into the software application (app) to tell them its decision, and its explanation. Later in the evening another cousin asks what it would say about them; you have left your ‘phone in the other room but after using the app about 15 times you believe you know what it will say. You ask for your cousin’s details and tell them your prediction of the app’s decision: it will say they are under the limit. Is your prediction about the app’s decision accurate? And how helpful was the explanation of its decision for understanding it?

We examine in four experiments how people reason about counterfactual explanations for an AI’s decisions. Despite extensive psychological research on how people create counterfactual alternatives about how things could have turned out differently, few studies have examined the use of counterfactuals as explanations for others’ decisions (for a review, see Byrne, 2016). People tend to create counterfactual explanations in daily life after something bad happens (e.g., Kahneman & Tversky, 1982; Ritov & Baron, 1995; Roese, 1997). They often use counterfactuals to justify or defend their own past actions that led to something bad, in situations ranging from political discourse to accident safety reports (e.g., Catellani & Covelli, 2013; Markman et al., 2008a, 2008b; Morris & Moore, 2000). Accordingly, counterfactuals amplify or deflect judgements of blame, moral responsibility, or legal culpability (e.g., Branscombe et al., 1996; Malle et al., 2014; Parkinson & Byrne, 2017; Tepe & Byrne, 2022). People often rely on them to excuse their own poor performance (e.g., Ferrante et al., 2013; Markman & Tetlock, 2000; McCrea, 2008). When such explanations focus on how things could have turned out better, for example, *“I would have got high marks if I had studied more”*, they help people to prepare for the future, providing a ‘roadmap’ for intentions and plans, for example, *“I will study more”,* enabling them to learn from mistakes and prevent the recurrence of bad outcomes (e.g., De Brigard et al., 2013; Dixon & Byrne, 2011; Markman et al., 2008a, 2008b; Roese & Epstude, 2017; Smallman & Roese, 2009).

Counterfactual explanations tend to focus on one’s own decisions, and little is known about how people understand such explanations for others’ decisions. Yet currently hundreds of AI decision support systems provide human users with counterfactual explanations for the AI’s decisions. People are increasingly provided with AI decisions in many areas of daily life, from health to finances, job recruitment to holiday choices. Although early machine learning systems were readily interpretable, the increased reliance on deep neural networks trained on vast arrays of data has resulted in successful AI systems that are hard-to-understand “black boxes” (e.g., Barredo Arrieta et al., 2019). The goal of eXplainable AI (XAI) is to develop algorithmic techniques to provide automated explanations, to improve the interpretability of an AI system and its decisions (for reviews, see Karimi et al., 2020; Keane et al., 2021). People are legally entitled to such explanations, and explanations may also improve their trust and willingness to accept AI decisions (e.g., Hoffman et al., 2018; Wachter et al., 2017). Some XAI techniques attempt to explain the whole AI system, whereas others attempt to justify the decision (e.g., Karimi et al., 2020; Verma et al., 2020). Recently, there has been an explosion of interest in XAI in counterfactual explanations, i.e., explanations that describe how the AI’s decision would have been different, if it had received different input information about some key feature. A frequently used example is an automated decision by a banking AI system to refuse a customer’s loan application, explained by the counterfactual, “*if you had asked for a lower amount, your loan application would have been approved”* (e.g., Dai et al., 2022; Warren et al., 2023). Over 100 distinct computational methods for generating counterfactuals using different automated approaches have been proposed (see Karimi et al., 2020; Keane et al., 2021). These alternative proposals have claimed the particular algorithmic method generates “good” counterfactuals for humans, using various criteria, for example, counterfactuals said to be plausible, actionable, proximal, sparse or diverse (e.g., Karimi et al., 2020; Keane & Smyth, 2020; Wachter et al., 2017; Warren et al., 2022). But crucially, such XAI claims about the usefulness of counterfactual explanations of AI decisions for human users are based on intuition rather than psychological evidence; a recent review identified fewer than 20% of almost 120 XAI counterfactual papers conducted any test of how human users understood the explanations (Keane et al., 2021).

The XAI interest in counterfactual explanations has been driven partly by their relation to causal explanations (Byrne, 2019; Miller, 2019). Suppose the app you had downloaded about alcohol and driving gave you a causal explanation instead: *You are under the limit because you drank on a full stomach*. Would the accuracy of your prediction of its decisions be better given the counterfactual or the causal explanation? Counterfactual explanations are similar to causal ones in a number of ways. Counterfactual explanations depend on identifying the relations between events, especially causal, intentional, or deontic relations, and the link between counterfactual and causal reasoning has received particular attention (e.g., Gerstenberg et al., 2021; Lewis, 1973; Lucas & Kemp, 2015; Meder et al., 2010; Spellman & Mandel, 1999). When participants are given or can generate a counterfactual, for example, “*I would have got high marks in the exam if I had had extra time*”, their judgements increase that the events are causally related, for example, “*I did not get high marks in the exam because I did not have extra time*” (e.g., McCloy & Byrne, 2002; see also Lagnado et al., 2013). But counterfactual explanations also differ from causal ones in a number of ways. Their content sometimes diverges; when people construct causal explanations, they tend to focus on strong causes that co-vary with an outcome, for example, a drunk driver caused the crash, whereas when they construct counterfactuals they tend to focus on background conditions that could have prevented it, for example, the crash wouldn’t have happened if the protagonist had driven home a different way (Mandel & Lehman, 1996).

Even when counterfactual and causal explanations have the same content, their mental representations differ in important ways. People create a counterfactual by “undoing” some aspects of their simulation of what happened, often to add something new (e.g., Kahneman & Tversky, 1982a; Roese & Epstude, 2017). They envisage at least two possibilities to compare the imagined alternative to what actually happened (e.g., Byrne, 2005). When they understand a factual conditional in the indicative mood *“if I had extra time, I got high marks”,* they initially envisage a single possibility, *“I had extra time and I got high marks”*, and although they know there may be alternatives consistent with the conditional, they do not think about them at the outset (Johnson-Laird & Byrne, 2002). Similarly, for a causal assertion, *“Because I had extra time I got high marks”*, they initially envisage a single possibility, and they can think about alternative possibilities subsequently (e.g. Frosch & Byrne, 2012; Johnson-Laird & Khemlani, 2017). But for a counterfactual conditional in the subjunctive mood *“if I had had extra time I would have got high marks”,* from the very outset people envisage not only the conjecture, *“I had extra time and I got high marks”*, they also recover the known or presupposed facts, *“I did not have extra time and I did not get high marks”* (Byrne, 2017). Hence, when people hear a counterfactual they look at images corresponding to both the alternative to reality and the facts, whereas when they hear a causal assertion they look at images corresponding just to the facts (Orenes et al., 2022). They also make more inferences from counterfactuals than factual assertions (e.g., Byrne & Tasso, 1999). On this dual possibility theory, counterfactuals provide richer information than causal assertions, because they are mentally represented at the outset by more possibilities; but they also require more cognitive resources, again because they are represented by more possibilities. Hence in diary studies, people spontaneously create fewer counterfactual explanations than causal ones (e.g., McEleney & Byrne, 2006). Generally, people prefer simple rather than complex causal explanations (e.g., Keil, 2006; Lombrozo, 2007; Quinn et al., 2021).

In artificial intelligence and machine learning, “causal explanation” is often reserved for scenarios in which a causal model is available. Hence some XAI studies refer instead to “rule-based explanations” or “if…then” rule explanations (e.g., Lage et al., 2019; van de Waa et al., 2021). However, in line with the typical terminology used in the psychology of explanation, we use “causal explanation” to refer to assertions such as “A because of B”, or “A happened because B happened” (see Keil, 2006; Kirfel et al., 2022; Johnson-Laird & Khemlani, 2017; Lombrozo, 2007). The few XAI human user studies of counterfactual explanations suggest counterfactuals can help users predict what an AI system will do (e.g., Lage et al., 2019; Lucic et al., 2020; van der Waa et al., 2021), and also improve their trust and satisfaction with the AI system (e.g., Förster et al., 2021; Kenny et al., 2021; Lucic et al., 2020; see also Hoffman et al., 2018). But although people’s evaluation of their satisfaction and trust in an AI system is higher for one that provides counterfactual explanations than causal ones, their accuracy in predicting the AI’s decisions is helped equally by both sorts of explanation (Warren et al., 2023). The dissociation has raised concerns about ethical explanation strategies in AI: if an explanation type is preferred by human users but has little added impact on their knowledge of the system, it could lead them subjectively to trust an app’s decision without objectively understanding it (Warren et al., 2023). We aim to examine further whether people find counterfactual explanations more helpful than causal ones. We will examine participants’ subjective judgements using the more sensitive measure of their evaluation of the helpfulness of each explanation of an AI’s decision as each one is presented, rather than by a general set of questions about overall explanation satisfaction administered at the end of the experiment (as in Warren et al., 2023). We will also examine their accuracy in predicting the AI’s decisions when their attention has been drawn to each explanation in this manner.

We test not only a familiar domain, alcohol and driving, but also an unfamiliar one, chemical safety. Suppose, instead of the blood-alcohol app, you have a college job for the summer in a chemical lab, and your employer has provided an AI decision support system to guide you in what chemicals are safe for you to handle. You enter the information it requires: occupational exposure limit, pH, exposure duration, air pollution rating, and PNEC rating, and it provides you with a decision: *Chemical 83220 is safe*. It also provides you with an explanation for its decision: *Chemical 83220 would have been deemed unsafe if it had had a longer exposure duration.* You use the app about 15 times on your first day and then you are faced with another chemical in a different room from where the device with the AI app is located. You think about this chemical’s details and come to a prediction of the app’s decision: it will say the chemical is safe. Is your prediction accurate about the app’s decision? And does the task of predicting the chemical safety app’s decision seem harder than predicting the alcohol and driving app’s decision? For a familiar domain, it is likely people already have beliefs about what prediction the AI system will make, for example, about whether someone who has drunk 10 units of alcohol is over the legal limit to drive, whereas in an unfamiliar domain, they may have few beliefs about what prediction the AI system will make, for example, about whether a chemical with a pH of 10 units is unsafe to handle.

People make very different inferences with familiar content compared to unfamiliar content. Many hundreds of experiments have shown that people make more accurate inferences about how to test a rule when it is about familiar content, for example, “*if an envelope is sealed then it has a 90 cent stamp*” compared to unfamiliar content, for example, “*if a card has a vowel on one side then it has an even number on the other side*” (Wason, 1968; Johnson-Laird, et al, 1972; for a review, see Nickerson, 2015). Familiar content elicits people’s prior beliefs (e.g., Evans & Over, 2004; Oaksford & Chater, 2007) and it makes counterexamples to putative conclusions readily available (Sperber et al., 1995). Knowledge of a domain modulates people’s models of a situation, enabling them to envisage more possibilities, and to eliminate some possibilities from further consideration (e.g., Johnson-Laird & Byrne, 2002; Ragni et al., 2018). Hence we expect participants will make more accurate predictions for a familiar domain compared to an unfamiliar one. We examine whether they consider counterfactual explanations more helpful than causal ones not only for a familiar domain, but also for an unfamiliar one. In a familiar domain people will be readily able to envisage the dual possibilities for a counterfactual explanation, which correspond to their prior knowledge, so they will have access to more information than for a causal explanation; we test whether in an unfamiliar domain they are as readily able to envisage such dual possibilities. Familiarity of domain is relevant to XAI since in some situations, for example, health or holiday choices, people may have some prior beliefs, whereas in other domains, for example, finance or job recruitment, they may not; yet familiarity is relatively unexplored in the study of counterfactual explanations, whether in psychology or in XAI.

**Fig. 1:**
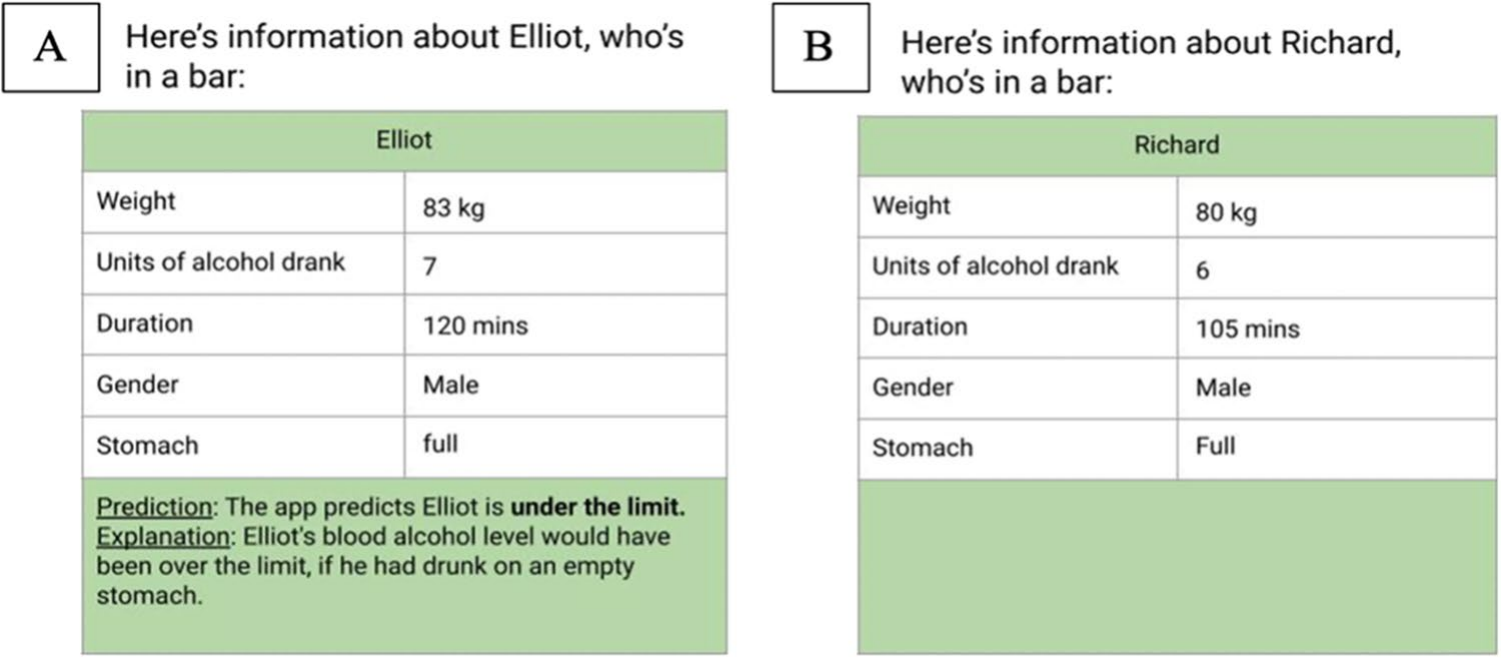
An example of a case presented in the first part of Experiment 1 in **A** (for the familiar domain with a counterfactual explanation); and a case presented in the second part of the experiment in **B**. (The cases are presented side-by-side for illustration, participants saw each case in isolation)

Our goal is to examine how people reason about counterfactual explanations of others’ decisions, using AI decisions as a test bed given their topicality. The approach in our four experiments was to provide participants with a set of diverse cases consisting of a variety of inputs provided to an AI system, the different decisions it made, and an explanation for why it made each decision; and to ask participants to judge how helpful the explanation was. An example of the tabular information given to participants, including the AI’s decision and a counterfactual explanation is provided in Fig. 1A. After participants gained experience with a set of cases, the decisions the AI system made, and the explanations for its decisions, we presented them with an entirely new set of different cases, this time with no information about the AI’s decision, and asked them to predict the AI’s decision, for example, whether it would decide the person was over the limit or under the limit. Figure 1B provides an example of the information in this prediction task.

The paradigm, or use-case, in which participants are first provided with trials to become familiar with an app’s decisions, before then being asked to make judgements such as predictions about the app’s decisions, was chosen to ensure that participants commence the prediction task from a similar baseline, and is consistent with proposals that the psychological impact of an explanation is knowledge change (e.g., Keane, 2023; Keil, 2006). Participants’ responses are likely to be based in part on their beliefs about blood alcohol level and driving limits, and in part on their recollection of the somewhat similar cases they considered in the first part of the experiment and the decisions the AI system made. Our interest is in whether participants’ predictions are affected differently by counterfactual explanations, for example, “*Elliot’s blood alcohol level would have been over the limit, if he had drunk on an empty stomach*”, or causal explanations, for example, “*Elliot’s blood alcohol level was under the limit, because he drank on a full stomach*”. The content of the explanations is the same, and the informational, memory, and response demands of the prediction task are the same, all that differs is whether the explanation is phrased as a conditional using the connective “if” and the subjunctive mood, or as a causal assertion using the connective “because” and the indicative mood.

We compared counterfactual to causal explanations for AI decisions in a familiar domain and an unfamiliar one; on participants’ predictions of an AI’s decisions (Experiment 1), and on their own decisions (Experiment 2); for an AI system that provides correct decisions and explanations, and one that provides incorrect ones (Experiments 3a and 3b). Since previous studies found trends of increased subjective preference and objective accuracy for counterfactual and causal explanations compared to control descriptions (Warren et al., 2023), we compared counterfactual and causal explanations directly to each other in our experiments.

A summary of the four main findings we will report is as follows: (a) Participants judged counterfactual explanations more helpful than causal ones in our first experiment, but counterfactuals did not improve the accuracy of their predictions of an AI’s decisions more than causals. (b) However, in our second experiment, counterfactuals improved the accuracy of participants’ own decisions more than causals. (c) In these two experiments, the AI’s decisions and explanations were correct and participants considered explanations more helpful and made more accurate judgements in the familiar domain than the unfamiliar one. (d) In our third and fourth experiments, the AI’s decisions and explanations were incorrect, and participants considered explanations less helpful and made fewer accurate judgements in the familiar domain than the unfamiliar one, whether they predicted the AI’s decisions or made their own decisions.

## Experiment 1: Predictions of an Artificial Intelligence (AI)’s decisions

Our first hypothesis was people will judge explanations for an AI’s decisions to be more helpful in a familiar than an unfamiliar domain, and they will be more accurate in predicting its decisions in the familiar than the unfamiliar domain. Our second hypothesis was people will judge counterfactual explanations more helpful than causal ones even when we measure explanation helpfulness by judgements after each explanation, rather than by a final satisfaction scale (as in Warren et al., 2023). We also test whether counterfactual and causal explanations help prediction accuracy equally even when participants’ attention is drawn to each explanation in this manner.

### Method

#### Participants

A g*power analysis indicated 171 participants were required to achieve 90% power for a two-tailed analysis of variance (ANOVA) with a medium size effect at *p* < 0.05. Participants in each experiment were recruited through Prolific, and paid £1.50 sterling; they were native English speakers from Ireland, Britain, America, Canada, Australia and New Zealand, who had not previously participated in related studies. The 177 participants included 139 women, 31 men, five non-binary people, and two people who preferred not to say; their average age was 24.9 years with a range of 18–56 years. They were assigned to four groups: Familiar counterfactual (n = 45), Familiar causal (n = 48), Unfamiliar counterfactual (n = 44), and Unfamiliar causal (n = 40). Participants were excluded prior to any data analysis if they failed either of two attention checks, or failed to correctly identify at least three of five features in a memory test, and accordingly, a further 24 participants were excluded. The experiments received prior approval from the Trinity College Dublin School of Psychology ethics committee, reference SPREC102020-52.

#### Design and materials

The design was a 2 (familiarity: high, low) × 2 (explanation type: counterfactual, causal) between-participants design, and participants were assigned to one of four conditions. The dependent measures were judgements of explanation helpfulness, accuracy of predictions, and confidence in predictions.

In the first part of the experiment participants were presented with 16 cases (see Fig. 1a). Each case consisted of the input provided to an AI system, the decision it made, and an explanation for why it made the decision. The cases were presented in a different randomised order for each participant. For each case, participants were asked to rate the helpfulness of the explanation, i.e., they responded to, “*This explanation was helpful*” by ticking a 1–5 Likert-type scale (labelled: strongly disagree, disagree, neutral, agree, strongly agree). The 16 cases are presented in the Online Supplementary Materials (OSM).

In the second part, participants were presented with 16 different cases (see Fig. 1b). Each case consisted only of the input provided to the AI system, without its decision or any explanation. The cases were presented in a different randomised order for each participant. Participants were asked to predict the AI’s decision, responding to the prompt, *“Based on the information provided, I believe the app’s prediction for this person/chemical will be”* by selecting from the binary options of “*over the limit*/*under the limit*” in the familiar condition, or “*safe/not safe*” in the unfamiliar condition. They were also asked to judge how confident they were in their prediction on a 1–5 Likert-type scale (labelled: not at all confident, not very confident, neither, fairly confident, very confident). The 16 cases are presented in the OSM. Participants received two attention check items, one in each part of the experiment, also presented in tabular form visually identical to the target cases, but participants were asked to indicate the value of one of the input features.

To familiarise participants with the response options, before beginning the experimental trials, they completed one example trial for the first part of the experiment, and a second example trial for the second part. Hence, participants knew from the outset they were going to judge the helpfulness of explanations, and then predict the AI’s decisions.

##### *SafeLimit*

Participants in the familiar condition were instructed they would be testing a new app named *SafeLimit* (adapted from Warren et al., 2023). They were told the app was designed to predict whether or not a person would be over the legal limit to drive, based on five features the app analysed: the person’s weight, units of alcohol, duration of drinking, gender, and stomach fullness. The cases for the *SafeLimit* system were taken from an AI case-base used to estimate an individual’s blood alcohol content (BAC) using the Widmark formula (Posey & Mozayani, 2007). For the purpose of this study, individuals with BAC ≥ 0.08% were classified as over the legal limit to drive and the selection of the 34 cases used as experimental materials was limited to cases with BAC proximal to the decision boundary of being over or under the limit (0.06 < BAC < 0.09). The outcome for half of the selected cases was over the limit and for the other half was under the limit. Further information on the selection of cases is given in the OSM.

##### *Chemsafe*

**Fig. 2:**
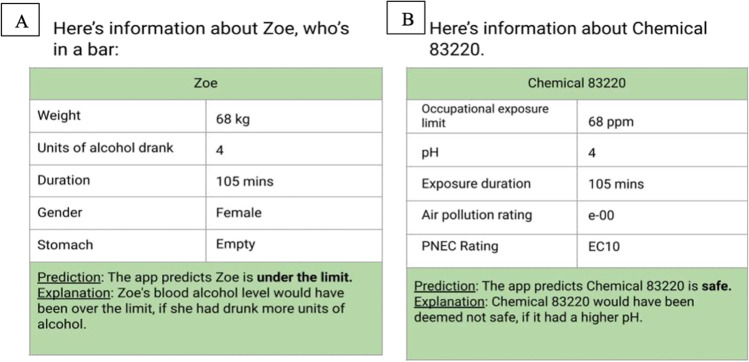
The same case presented in the familiar blood-alcohol condition in **A**, and the unfamiliar chemical safety condition in **B**

Participants in the unfamiliar condition were instructed they would be testing a new app called *ChemSafe*. They were told the app was designed to predict whether or not a chemical would be safe to handle, based on five features the app analysed: occupational exposure limit, pH, exposure duration, air pollution rating, and PNEC rating. The cases for the *ChemSafe* app were created to be analogous to the blood alcohol cases and the same ones were used from the *SafeLimit* app with modifications to reduce familiarity, i.e., features from the BAC system were converted to chemical safety technical terms, while the values and case logic remained the same. The categorical features of gender (male/female), and stomach fullness (empty/full) were modified into categorical features of air pollution rating (e-01/e-00), and PNEC rating (EC10/EC50), and the continuous features of weight (kg), units (units), and duration (minutes) were modified into continuous features of occupational exposure limit (ppm), pH (units), and exposure duration (minutes). Figure 2 provides an example of a case from *SafeLimit* converted to *ChemSafe* (see Table S1 in the OSM for further information).

##### Explanations

Participants were given either counterfactual or causal explanations in the first part of the experiment. Participants in the counterfactual condition were given an explanation in the form of a conditional with the connective ‘if’ in the subjunctive mood, for example, “*Zoe's blood alcohol level would have been over the limit, if she had drunk more units of alcohol*”. Participants in the causal explanation condition were given a matched explanation with the same content but in the form of a causal assertion with the connective ‘because’ in the indicative mood, for example, “*Zoe's blood alcohol level was under the limit, because she drank few units of alcohol”*. Each explanation was based on one feature and to control for any effects of explanations about different features, participants were presented with four explanations related to each of the four features: weight, units of alcohol, duration, and stomach fullness (and for each of the four features, two outcomes were over the limit and two were under the limit). For the binary feature of stomach fullness, the counterfactual explanations referred to the opposite of the facts presented about it (i.e., *full/empty*). For the three continuous features of weight, units of alcohol, and duration, they referred to, for example, ‘more’ or ‘fewer’, for example, “*…if she had drunk fewer units of alcohol*”; the causal explanations referred to ‘many’ and ‘few’, for example, “*… because she drank many units of alcohol*.” Accordingly, counterfactual explanations used comparative descriptors whereas causal explanations used absolute ones. The difference is consistent with a counterfactual’s focus not only on the facts, for example, *she drank 8 units*, but also on an alternative to reality, *if she had drunk fewer units*; and a causal assertion’s focus on the facts, *she drank 8 units,* i.e., *many units* (see OSM).

At the end of the experiment, we gave participants two scales developed in the XAI literature to measure explanation satisfaction and trust in the AI system (Hoffman et al., 2018), used in previous studies (e.g., Warren et al., 2023). Their results were broadly consistent with the judgements of explanation helpfulness and so for brevity we report them in the OSM. Participants also completed a memory check question: to identify five features used by the app, by selecting them from a list of ten features (see OSM).

#### Procedure

Prolific users who consented to participate were provided with a link to the online experiment, presented via Alchemer. The experiment took approximately 15 min to complete.

### Results and discussion

The data for all the experiments are available via the Open Science Framework at https://osf.io/e7hjs/ In each experiment we carried out a 2 (familiarity: familiar vs. unfamiliar domain) × 2 (explanation type: counterfactual vs. causal) between-participants ANOVA, on explanation helpfulness judgements, prediction accuracy, and prediction confidence.

**Fig. 3:**
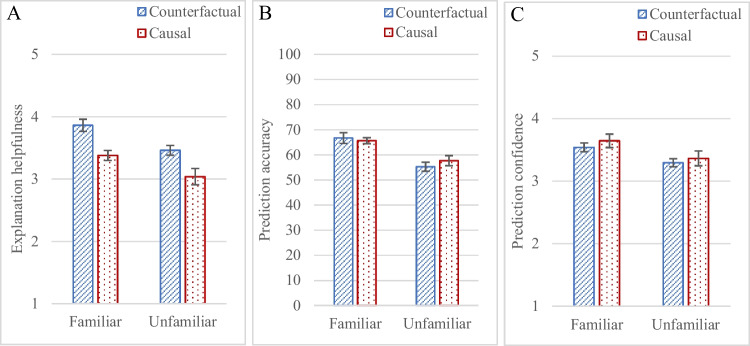
Mean explanation helpfulness judgements in **A** (on a 1–5 scale), percentage prediction accuracy in **B** (for 16 items in each condition), and mean prediction confidence in **C** (on a 1–5 scale), in Experiment 1. For prediction accuracy in **B**, chance accuracy is 50%; the average accuracy was 66% in the familiar conditions and 56% in the unfamiliar conditions. Error bars represent standard error of the mean

In the first part of the experiment, participants responded to, “*This explanation was helpful*” for each one of 16 explanations. They judged counterfactual explanations more helpful than causal explanations, F(1, 173) = 22.04, *p *<.001, η_p_^2^ = .11, and judged explanations more helpful in the familiar domain than the unfamiliar one, F(1, 173) = 14.73, *p* < .001, η_p_^2^ = .08; the two variables did not interact, F(1,173) = 0.10, *p* = .75, Fig. 3A. The same direction of difference occurred for most of the 16 items (see OSM for additional analyses of item consistency).

In the second part, participants predicted the AI’s decision for 16 new cases, by selecting from the binary options of “*over/under the limit*” (or “*safe/not safe*”). The prediction accuracy measure indicates the extent to which participants’ predictions aligned with the AI’s decisions. Participants made as many correct predictions whether they had been given counterfactual or causal explanations, F(1,173) = .14, *p* = .71; they made more correct predictions for the familiar domain than the unfamiliar one, F(1, 173) = 28.68, *p* < .001, η_p_^2^ = .14; the two variables did not interact, F(1,173) = .91, *p* = .34, see Fig. 3B. The same direction of difference was observed for most of the items (see OSM).

Participants were asked to judge how confident they were in their prediction for each one of the 16 cases. They were equally confident in their predictions whether they had been given counterfactual or causal explanations, F (1, 173) = .91, *p* = .34, they were more confident about their predictions for the familiar domain than the unfamiliar one, F(1, 173) = 8.54, *p* = .004, η_p_^2^ = .05; the two variables did not interact, F (1, 173) = .04, *p* = .84, Fig. 3C.

Participants judged counterfactual explanations more helpful than causal ones, but their prediction accuracy was helped equally by counterfactual as by causal explanations, and their confidence in their accuracy was the same whether given counterfactual or causal explanations. They judged explanations more helpful in the familiar domain than the unfamiliar one, their predictions were more accurate, and they were more confident in their decisions.

That people judge counterfactual explanations to be more helpful than causal ones suggests the informational benefit of the enriched mental representation of two possibilities for counterfactuals outweighs any cognitive costs of envisaging multiple models. Why then are people’s predictions of an AI’s decisions not helped more by counterfactual explanations than by causal ones? One potential explanation is the additional information in the dual representation of counterfactuals is of use when people reason about their own decisions, rather than another’s decisions. Our next experiment examines the effects of explanations on the accuracy of one’s own decisions.

## Experiment 2: Making one’s own decisions

Suppose once again you are at a family gathering and have had a few glasses of wine. Once again you have gained experience with the alcohol and driving app, and now you no longer have access to it. But suppose now you must decide whether you are prepared to drive home. Your task in this case is not simply predicting what the app would decide, instead it is to make your own decision about what is safe for you to do. The aim of Experiment 2 was to compare the effects of counterfactual and causal explanations for an AI’s decisions on participants’ own decisions.

Our focus on people’s own decisions arises because counterfactuals are often personal, i.e., people generate episodic counterfactual thoughts about how events in their own lives could have turned out differently, often centred on their goals, i.e., to prepare for the future by forming intentions, for example, to prevent a bad outcome from occurring again (e.g., De Brigard et al., 2013; Ferrante et al., 2013; Roese & Epstude, 2017). Counterfactuals are influential in helping people make future decisions (O’Connor et al., 2014). In the experiment participants knew from the outset they were going to make decisions about their own safety to drive, or to handle chemicals (because once again they received at the outset a practice trial for each part of the experiment). The task helps ensure participants’ engagement with the AI’s decisions and explanations. In the previous experiment, participants attempted to predict the AI’s decisions about others, given knowledge of the AI’s past decisions about others; in this experiment they attempt to make a decision about themselves, given knowledge of the AI’s past decisions about others. The distinction between predicting an AI’s decisions about others’, and one’s own decisions, is relevant to XAI: AI systems have been shown to outperform humans in some domains (e.g., Bae et al., 2017), but in a variety of situations, for example, finance, medicine, or legislation, human users may ultimately decide whether or not to follow the AI’s decision. For example, a loan applicant may decide whether or not to apply for a lower loan, or a doctor may decide whether or not to follow a recommendation for a patient’s treatment.

Our hypothesis was that when participants make their own decisions, they will judge counterfactual explanations more helpful than causal ones, and their own decisions will be more accurate given counterfactual explanations than causal ones (i.e., the dissociation between subjective and objective measures will be eliminated). We expect once again their decisions will be more accurate when they are familiar with the domain than unfamiliar with it.

### Method

#### Participants

The 173 participants included 122 women, 46 men, four non-binary people, and one person who preferred not to say; their average age was 36.2 years with a range of 18–77 years. They were assigned to four groups, Familiar counterfactual (n = 41), Familiar causal (n = 44), Unfamiliar counterfactual (n = 43), and Unfamiliar causal (n = 45). The participants were a new set who had not taken part in Experiment 1. Prior to any data analysis a further 36 participants were excluded because they failed one of the two attention checks, or the memory test; a further 49 participants were excluded because they indicated in response to two additional questions they had strong beliefs against ever drinking and driving, or ever handling unsafe chemicals (see below).

#### Design, materials and procedure

The design, materials and procedure were the same as the previous experiment, with two exceptions. The first exception was in the second part of the experiment, participants were asked to imagine the data provided was about themselves, and to make a decision, responding to the prompt, *“Based on the information provided, I would be prepared to”,* by selecting from the binary options of ‘*drive*/*not drive*’ in the familiar condition, or ‘*handle/not handle*’ the chemical in the unfamiliar condition (see OSM). The second exception was the addition of two extra questions at the end, on participants’ own beliefs about drinking and driving, or about handling chemicals (see OSM). It was decided to exclude any participants who agreed strongly (5 on the 1–5 scale) that people who had even one drink should not drive (or people should never handle unfamiliar chemicals), or disagreed strongly (1 on the 1–5 scale) that people under the legal limit can drive (or people can handle chemicals deemed safe). Given participants were asked to make a decision about their own behaviour, the questions were included in case participants with strong beliefs responded to every case with the same decision (e.g., do not drive).

### Results and discussion

**Fig. 4:**
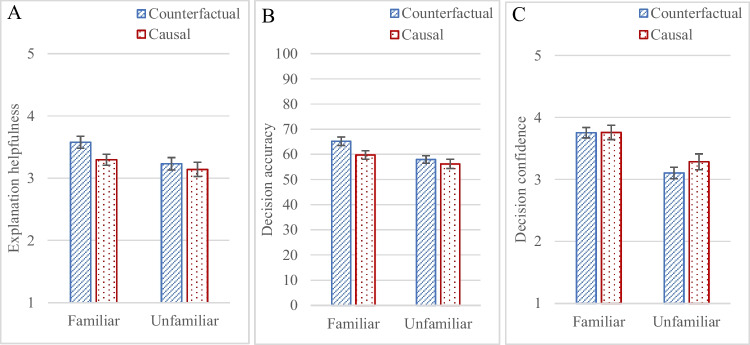
Mean explanation helpfulness judgements in **A** (on a 1–5 scale), percentage decision accuracy in **B** (for 16 items), and mean decision confidence in **C** (on a 1–5 scale), in Experiment 2. For decision accuracy in **B**, chance accuracy is 50%; the average accuracy was 63% in the familiar conditions and 57% in the unfamiliar conditions. Error bars represent standard error of the mean

The first part of Experiment 2 was identical to the first part of Experiment 1, and so we expected to replicate the findings for explanation helpfulness judgements. Participants judged counterfactual explanations somewhat more helpful than causal ones, although the difference did not reach significance, F(1, 169) = 3.379,* p* = .068, η_p_^2^ = .02; they judged explanations more helpful in the familiar domain than the unfamiliar one, F(1, 169) = 6.19,* p* = .014, η_p_^2^ = .035; the two variables did not interact, F(1, 169) = 0.88,* p* = .35, see Fig. 4A. The same direction of difference was observed for most of the items (see OSM).

The decision accuracy measure indicates the extent to which participants’ own decisions aligned with the AI’s decisions. Participants’ decisions were more accurate when they had been given counterfactual rather than causal explanations, F(1, 169) = 4.61, *p* < .03, η_p_^2^ = .03; and more accurate in the familiar domain than the unfamiliar one, F(1, 169) = 10.41, *p* < .002, η_p_^2^ = .06; the two variables did not interact, F(1, 169) = 1.22, *p* = .27, see Fig. 4B.

The same direction of difference between familiar and unfamiliar domains was observed for most items (see OSM). However, the same direction of difference between counterfactual and causal explanations was not observed for all items. Participants’ decisions were more accurate when they had been given counterfactual rather than causal explanations (more than 5% difference) for eight of the 16 items, but more accurate when they had been given causal rather than counterfactual explanations for six items, with no difference (greater than 5%) for two items. In testing the consistency of items further, participants’ decisions were more accurate for the eight items with bad outcomes than the eight items with good outcomes, F (1, 169) = 48.923, p < .0001, η_p_^2^=.224, in a 2 (explanation) × 2 (familiarity) × 2 (outcome: good – *under the limit/safe to handle*, vs. bad - *over the limit/unsafe to handle*) ANOVA with repeated measures on the third factor. Their decisions about items with good outcomes were more accurate when they had received counterfactual explanations rather than causal ones, t (171) = 3.482, p < .001, CI [-.23773, -.0657], but more accurate about items with bad outcomes when they had received causal explanations rather than counterfactual ones, t (171) = 2.196, p < .029, CI [.00822, .15413], in the decomposition of the interaction of outcome with explanation, F (1, 169) = 10.594, p < .001, η_p_^2^=.059 (see OSM). 

Participants were equally confident in their predictions whether they had been given counterfactual or causal explanations, F(1, 169) = .75, *p* = .39; they were more confident in decisions they made in the familiar domain than the unfamiliar one, F(1, 169) = 27.37, *p* < .001, η_p_^2^ = .14; the two variables did not interact, F(1, 169) = .66, *p* = .42, see Fig. 4C.

Participants judged counterfactual explanations somewhat more helpful than causal ones, and they were more accurate in their own decisions when they were given counterfactual explanations rather than causal ones overall. Although participants’ preferences for different explanations did not correspond to accuracy differences in Experiment 1 (and in Warren et al., 2023), in this experiment, their preferences corresponded to their accuracy differences. The experiment shows that two reasonable explanation types can lead to different accuracy.

Moreover, participants’ decisions were more accurate about good outcomes when they had received a counterfactual explanation, for example, “*Elliot's blood alcohol level would have been over the limit, if he had drunk on an empty stomach”,* rather than a causal one, for example, “*Elliot's blood alcohol level was under the limit, because he drank on a full stomach”*. But their decisions were more accurate about bad outcomes when they had received a causal explanation, for example, “*Jarred's blood alcohol level was over the limit, because he drank on an empty stomach”*, rather than a counterfactual one, for example, “*Jarred's blood alcohol level would have been under the limit, if he had drunk on a full stomach”*. The pattern may reflect the greater potency of an explanation that explicitly refers to a bad outcome (e.g., *over the limit*): the counterfactual mentions the bad outcome when the real outcome was good, whereas the causal mentions the bad outcome when the real outcome was bad. The result suggests that counterfactual explanations may be particularly helpful for guiding future decisions when they have been given as explanations of good outcomes, at least when they provide a downward comparison to how the situation could have been worse; whereas causal explanations may be particularly helpful for guiding future decisions when they have been given as explanations of bad outcomes.

Experiments 1 and 2 examined explanations of an AI’s decisions that were correct. We turn now to examine explanations of an AI’s decisions that are incorrect.

## Experiments 3a and 3b: Incorrect AI decisions

Counterfactual explanations sometimes attempt to justify poor decisions, for example, a decision not to vaccinate (e.g., Baron & Ritov, 2004; Ferrante et al., 2013; McCrea, 2008), and the explanation itself may also be poor, for example, “*if I had received the vaccine, I would have become more ill from the virus*”. The aim was to examine the effects of explanations for an AI system that provided incorrect decisions and explanations. AI systems generally have a high probability of accuracy but nonetheless can make errors, and even small error rates undermine human trust in their decisions (e.g., Kenny et al., 2021). When an AI system makes an incorrect decision in a familiar domain, for example, someone who has drunk 10 units of alcohol over a short duration on an empty stomach is judged to be under the legal limit to drive, people are likely to identify the error. When they predict the AI’s decisions, they are confronted with a conflict between whether to rely on their memory of the information they gained about the AI’s decisions, or on their own prior knowledge. We expect their predictions of the AI’s decisions will be inaccurate as a result, in that they will not align with the AI’s decisions, because its decisions conflict with their own knowledge. We also expect they will judge explanations of such incorrect decisions unhelpful in the familiar domain. An explanation such as *“Elliot was under the limit because he drank on an empty stomach”* presents a causal relationship that appears clearly implausible. In contrast, when an AI system makes an incorrect decision in an unfamiliar domain, for example, a chemical with a pH of 10 units with a short exposure duration and a PNEC rating of EC10 is safe to handle, participants will be less likely to spot the error since they have no prior knowledge to guide them. An explanation such as *“Chemical 37286 was safe to handle because it had a PNEC rating of EC10”* presents a causal relationship that may appear plausible. For an AI system that provides incorrect decisions and explanations, we hypothesise participants will judge explanations to be *less* helpful in the familiar domain than the unfamiliar one, and they will make *fewer* accurate predictions in the familiar domain than the unfamiliar one.

We presented participants with an AI system which made the opposite decision for each of the cases used in the previous experiments. Of course, an AI system that makes wholly incorrect decisions is an extreme situation which would never make it to market. We chose to present an AI system with all incorrect decisions because the cases in the previous experiments had been selected to be close to the decision boundary, i.e., the decision was close to being its opposite. Hence, it provides the best opportunity for participants to detect the decisions are incorrect. It enables us to test whether their judgements about the helpfulness of explanations and their accuracy are affected by the correctness of the AI’s decisions. In Experiment 3a participants predicted the AI’s decisions and in Experiment 3b they made their own decisions.

### Method 

#### Participants

The 184 participants in Experiment 3a included 116 women, 63 men, four non-binary people, and one person who preferred not to say; their average age was 39.1 years with a range of 18–74 years. The 186 participants in Experiment 3b included 122 women, 63 men, and one person who preferred not to say; their average age was 39 years with a range of 19–72 years. The participants in Experiment 3a were assigned to four groups: Familiar counterfactual (n = 48), Familiar causal (n = 49), Unfamiliar counterfactual (n = 43), and Unfamiliar causal (n = 44). The different set of participants in Experiment 3b were also assigned to four groups: Familiar counterfactual (n = 49), Familiar causal (n = 43), Unfamiliar counterfactual (n = 47), and Unfamiliar causal (n = 47). The participants in Experiments 3a and 3b were a new set who had not taken part in the previous experiments. Prior to any data analysis, a further 24 participants were excluded from Experiment 3a, and a further 55 participants were excluded from Experiment 3b, for failing one of the two attention checks or the memory test. In addition, another 59 participants from Experiment 3b were excluded because they indicated they had strong beliefs against ever drinking and driving, or ever handling potentially unsafe chemicals.

#### Design, materials and procedure

**Fig. 5:**
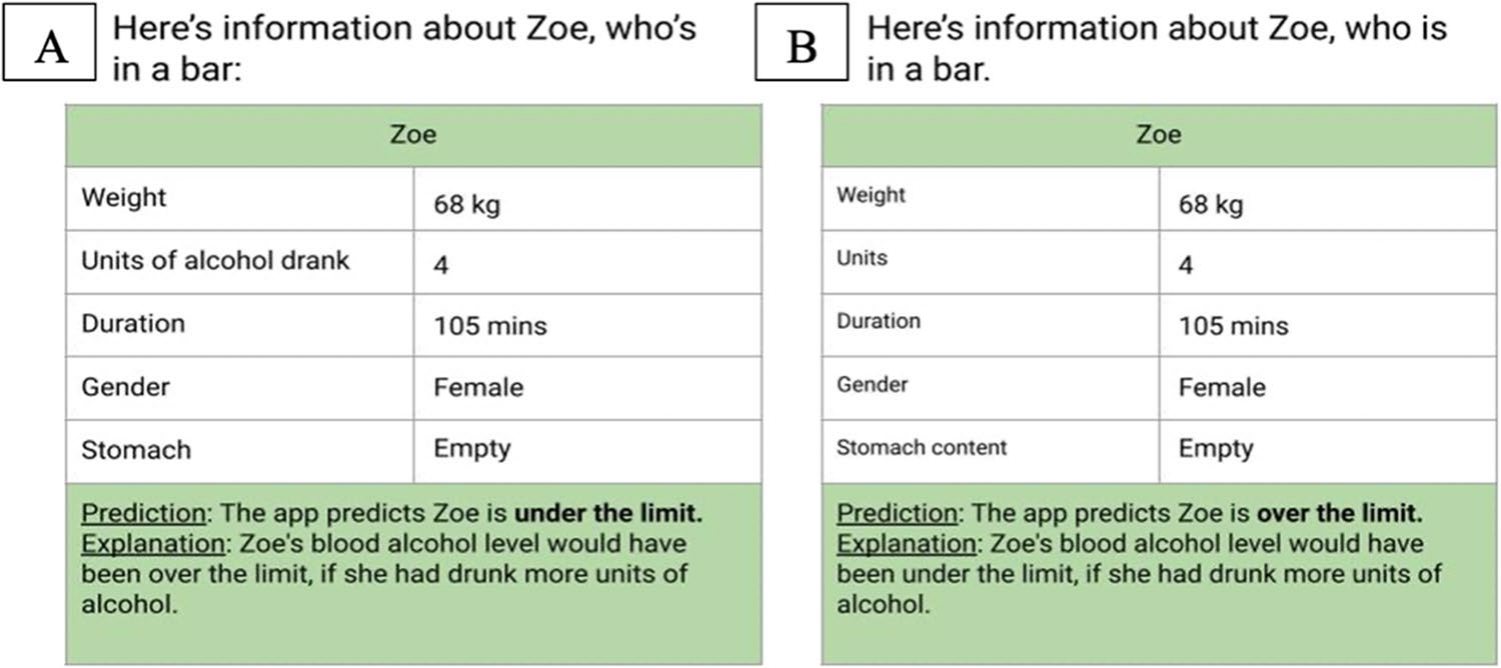
An example of a case with the correct artificial intelligence (AI) system decision and explanation used in Experiments 1 and 2 in **A**, and the same case with an incorrect AI system decision and explanation used in Experiments 3a and 3b in **B**

The design and procedure of each experiment were the same as the previous experiments. The materials were different. The experiments presented incorrect AI system outputs (i.e., the app made an incorrect prediction according to the Widmark formula). The same cases from the previous experiments were used, the features and their values remained the same, but the decision the AI system made and the explanation provided were modified. Incorrect outputs were generated by inverting the original prediction (‘under the limit’ to ‘over the limit’, ‘safe’ to ‘not safe’, and vice versa). The explanations provided were modified to be incorrect too, in that the relationship between the feature and the outcome was inverted, for example, an explanation about units of alcohol indicated fewer units of alcohol would lead to the person being over the limit, see Fig. 5.

The adjustments made to the chemical app to create incorrect outputs were analogous to those made to the blood alcohol app. Since the chemical app is entirely fictional, there is no true sense in which its predictions are correct or incorrect, and the adjustments were made to ensure it could be used as a control unfamiliar domain.

## Results and discussion

**Fig. 6:**
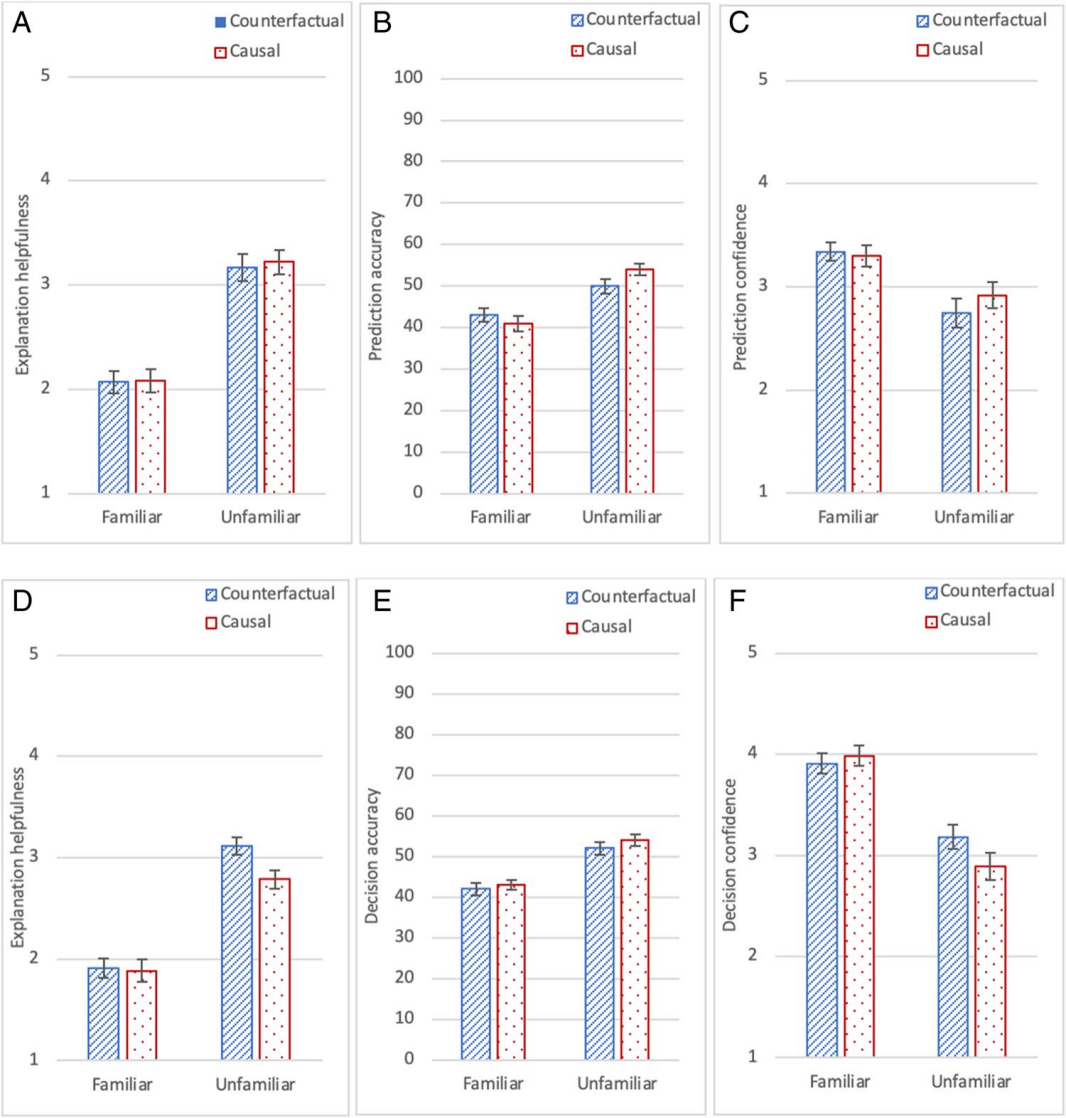
Mean explanation helpfulness judgements in **A** (on a 1–5 scale), percentage prediction accuracy in **B** (for 16 items), and mean prediction confidence in **C** (on a 1–5 scale), in Experiment 3a for an incorrect AI system. For prediction accuracy in **B**, chance accuracy is 50%; the average prediction accuracy was 42% in the familiar conditions and 52% in the unfamiliar conditions. Mean explanation helpfulness judgements in **D** (on a 1–5 scale), percentage decision accuracy in **E** (for 16 items), and mean decision confidence in **F** (on a 1**–**5 scale), in Experiment 3b for an incorrect AI system. For decision accuracy in **E**, chance accuracy is 50%; the average decision accuracy in Experiment 3b was 43% in the familiar conditions and 53% in the unfamiliar conditions. Error bars represent standard error of the mean

When presented with incorrect system outputs, participants judged explanations as *less* helpful for the familiar domain than the unfamiliar domain, in Experiment 3a, F(1, 180) = 92.54, p < .001, η_p_^2^ = .34, and Experiment 3b, F(1, 182) = 119.94, p < .001, η_p_^2^ = .40; they judged counterfactual and causal explanations equally helpful, in Experiment 3a, F (1, 180) = .02,* p* = .89, and Experiment 3b, F(1, 182) = 3.46, p = .06, η_p_^2^ = .02; the two variables did not interact, in Experiment 3a, F (1, 180) = .06, *p* = .81, or Experiment 3b, F(1, 182) = 2.46, p = .12, see Fig. 6A and D.

The accuracy measure indicates the extent to which participants’ predictions, or their own decisions, aligned with the AI’s (incorrect) decisions. When presented with incorrect system outputs, participants made *fewer* accurate predictions or own decisions, i.e., predictions or own decisions that were the same as the AI’s incorrect decisions, for the familiar domain than the unfamiliar one, in Experiment 3a, F(1, 180) = 34.95 , *p* < .001, η_p_^2^ = .16, and in Experiment 3b, F(1, 182) = 55.62, *p *< .001, η_p_^2^ = .23; their predictions or own decisions were equally accurate whether they had been given counterfactual or causal explanations, in Experiment 3a, F (1, 180) = .16, *p* = .69, and Experiment 3b, F(1, 182) = .84, *p* = .36; and the two variables did not interact, in Experiment 3a, F (1, 180) = 2.48, *p* = .12, or Experiment 3b, F(1, 182) = .00, *p* = .99, see Fig. 6B and E.

Participants were more confident about their predictions or own decisions for the familiar domain than the unfamiliar domain, in Experiment 3a, F(1,180) = 17.26, *p* < .001, η_p_^2^ = .09, and Experiment 3b, F(1, 182) = 63.41, *p* < .001, η_p_^2^ = .26; they were equally confident whether they had been given counterfactual or causal explanations, in Experiment 3a, F (1, 180) = .33, *p* = .57, and Experiment 3b, F(1, 186) = .89, *p* = .35; the two variables did not interact, in Experiment 3a, F (1, 180) = .86, p = .36, or in Experiment 3b, F(1, 182) = 2.51, *p* = .12, see Fig. 6C and F.

For an AI that provides incorrect decisions and explanations, participants judged explanations to be less helpful in the familiar domain than the unfamiliar one; their predictions or own decisions were less accurate in the familiar domain than the unfamiliar one, i.e., less aligned with the AI’s incorrect decisions, and they remained more confident in their predictions or own decisions in the familiar domain than the unfamiliar one. Participants’ confidence in their judgements was high despite the accuracy of their predictions being low. They were asked to judge how confident they were in their prediction or decision and hence their judgement likely reflects their confidence in their own knowledge of the domain, rather than their confidence in their understanding of the AI system. When an AI system makes incorrect decisions in the familiar domain, participants rely on their own knowledge when they predict the AI’s decisions or make their own decisions, rather than rely on their memory of the information they gained about the AI’s decisions in the first part of the experiment. For incorrect decisions of an AI system there was no subjective preference for counterfactual explanations over causal ones, and no increase in accuracy given counterfactual explanations rather than causal ones, for predictions or own decisions.

## General discussion

People often create counterfactual explanations for their past decisions. Our experiments show they judge counterfactual explanations for an AI’s decisions, for example, “*He would have been over the limit if he had drunk on an empty stomach*”, more helpful than causal ones, for example, “*He was under the limit because he drank on a full stomach*”, for familiar and unfamiliar domains, for correct AI decisions, see Table 1.

**Table 1:** A summary of the results of the four experiments (> indicates participants’ judgements or accuracy were greater, for example, familiar > unfamiliar)

	Experiment 1	Experiment 2	Experiment 3a	Experiment 3b
*Participants’ Judgements:*	Predictions	Decisions	Predictions	Decisions
*AI system outputs:*	Correct	Correct	Incorrect	Incorrect
Explanation Helpfulness	Familiar	Familiar	Familiar	Familiar
	>	>	<	<
	Unfamiliar	Unfamiliar	Unfamiliar	Unfamiliar
	Counterfactual	Counterfactual	Counterfactual	Counterfactual
	>	≥ *	=	=
	Causal	Causal	Causal	Causal
Accuracy	Familiar	Familiar	Familiar	Familiar
	>	>	** < **	<
	Unfamiliar	Unfamiliar	Unfamiliar	Unfamiliar
	Counterfactual	Counterfactual	Counterfactual	Counterfactual
	=	>	=	=
	Causal	Causal	Causal	Causal
Confidence	Familiar	Familiar	Familiar	Familiar
	>	>	>	>
	Unfamiliar	Unfamiliar	Unfamiliar	Unfamiliar
	Counterfactual	Counterfactual	Counterfactual	Counterfactual
	=	=	=	=
	Causal	Causal	Causal	Causal

^*^ p = .068

The accuracy of their predictions of the AI’s decisions was the same whether they were given counterfactual or causal explanations (in Experiment 1). Strikingly, the accuracy of their own decisions, i.e., whether their decisions aligned with the AI’s decisions, was greater when they were given counterfactual rather than causal explanations (in Experiment 2). Their decisions were more accurate about good outcomes when they had been given a counterfactual, for example, “*He would have been over the limit if he had drunk on an empty stomach*”, rather than a causal, for example, “*He was under the limit because he drank on a full stomach*”; but their decisions were more accurate about bad outcomes when they had been given a causal, for example, “*He was over the limit because he drank on an empty stomach*”, rather than a counterfactual, for example, “*He would have been under the limit if he had drunk on a full stomach*”. Explanations that focus on a bad outcome, for example, *over the limit*, whether as a potential alternative (*“he would have been over the limit if…”*) or as a real occurrence (*“he was over the limit because…”*) improve the accuracy of their decisions.

Participants were equally accurate in their predictions of the AI’s decisions in Experiment 1 whether they were given counterfactual or causal explanations, despite their judgements of counterfactual explanations as more helpful than causal ones. The dissociation of subjective and objective measures occurred even when participants’ judged the helpfulness of each explanation individually (cf. Warren et al., 2023), and for both the familiar and unfamiliar domains.

However, participants were more accurate in their own decisions given counterfactual explanations than causal ones overall, in Experiment 2, in line with their tendency to consider counterfactual explanations somewhat more helpful than causal ones. The result somewhat ameliorates the ethical concerns over the dissociation between subjective and objective measures. The dissociation does not occur with personally engaging objective measures, for example, decision accuracy. The finding is consistent with counterfactuals as goal-directed towards decisions about what to do in the future (e.g., O’Connor et al., 2014; Roese & Epstude, 2017; see also Ferrante et al., 2013). It suggests the cognitive costs of representing dual possibilities for counterfactuals are outweighed by the benefits of available explicit information (e.g., Byrne, 2005, 2017; Orenes et al., 2022). If people prefer counterfactual to causal explanations, and they improve their own decision accuracy more, then they may indeed be useful for XAI. However, any advantage of counterfactual explanations over causal ones is moderated by the decision outcome: explanations that focus on a bad outcome, for example, being over the limit, whether as a potential alternative in a counterfactual explanation, or as a real occurrence in a causal explanation, may engage participants most.

People sometimes create counterfactual explanations to justify poor decisions, and their counterfactual explanations are also sometimes inaccurate. Little psychological research has been directed at the accuracy of counterfactual explanations. How people distinguish true counterfactuals from false ones (e.g., Byrne & Johnson-Laird, 2020), or probable from improbable ones (e.g., Over et al., 2007; Sloman & Lagnado, 2005), depends on their knowledge and ability to search for counterexamples. For the incorrect AI decisions and explanations in Experiments 3a and 3b, there was no subjective preference for counterfactual explanations over causal ones, and no objective differences in accuracy of predictions or own decisions. Counterfactual explanations for AI decisions may need to be calibrated to the error potential of the AI system. Future research on their impact for AI systems with different frequencies of incorrect decisions, or for incorrect decisions paired with correct explanations, is warranted. Explanations that reveal an incorrect causal relationship may be useful in enabling people to detect errors in an AI system’s decisions, at least in a familiar domain.

Familiarity with a domain has widespread effects on human reasoning (Nickerson, 2015). Psychological studies have distinguished episodic counterfactuals about people’s own lives, and semantic counterfactuals about hypothetical scenarios (Beck, 2020; De Brigard et al., 2013; Roese & Epstude, 2017). The effects of familiarity, i.e., domain knowledge or expertise, or a lack of it, on counterfactual thinking has not been widely explored in XAI (for a review, see Kenny et al., 2021). Participants judged explanations more helpful, and made more accurate predictions and own decisions, when they reasoned about an AI system in a familiar domain than one in an unfamiliar domain, when the AI system made correct decisions. Explanations in XAI may need to be calibrated to the domain knowledge of the human user and future research to examine other examples of familiar and unfamiliar domains, for example, finance, legislation, or medicine, and different levels of familiarity with a domain, would be fruitful. Moreover, when decisions and explanations provided by an AI system were incorrect, participants judged explanations as *less* helpful in the familiar domain than the unfamiliar one, indicating they were readily able to identify incorrect decisions and explanations in the familiar domain. One implication is intolerance for incorrect AI decisions may be pronounced in familiar domains.

Participants made *fewer* accurate predictions in the familiar domain than in the unfamiliar one, i.e., predictions aligned with the AI’s decisions, when the AI’s decisions were incorrect. Human users of an AI system may believe it is attempting to reach a correct decision and is capable of doing so – their prediction of the AI’s decision was not based on its previous incorrect decisions; instead, they predicted it would make a decision based on their own understanding of the domain. The use of an XAI system often precedes a human decision, for example, an AI recommendation to reject a loan with the explanation *“if the client had asked for a lower amount, their loan application would have been approved”* may be evaluated by a bank clerk tasked with approving or rejecting a loan. Users of an XAI system may elect to make decisions based on their own knowledge rather than following an AI’s recommendations. Nonetheless, counterfactual explanations are persuasive for human users of an AI system – people prefer them to causal explanations, and they increase the alignment of their own decisions to the AI’s decisions, for example, about good outcomes, in familiar and unfamiliar domains.

### Supplementary Information

Below is the link to the electronic supplementary material.

Supplementary file1 (DOCX 4.78 MB)
