# Supplementary material for: How people reason with counterfactual and causal explanations for Artificial Intelligence decisions in familiar and unfamiliar domains
Source: Mem Cognit. 2023 Mar 24;51(7):1481–96. doi: 10.3758/s13421-023-01407-5 (PMC10520145; doi:10.3758/s13421-023-01407-5)
Supplement: Supplementary file 1 — Supplementary file1 (DOCX 4.78 MB) [file 13421_2023_1407_MOESM1_ESM.docx]

**Supplementary Materials**

*Materials for the familiar domain (SafeLimit)*

We provide (a) the 16 cases for the first part of the experiments with counterfactual explanations, (b) the same cases with causal explanations, (c) the different set of 16 cases for the prediction part, (d) an example of one used in the decision experiments.

1. ***The 16 cases with counterfactual explanations:***

| 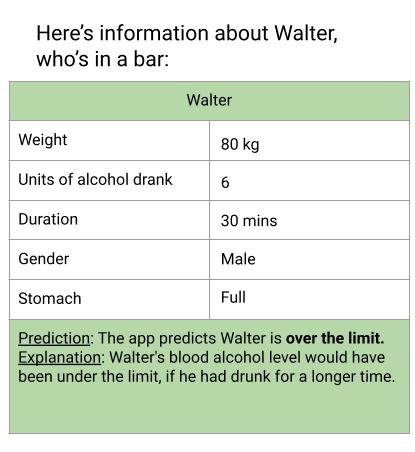 | 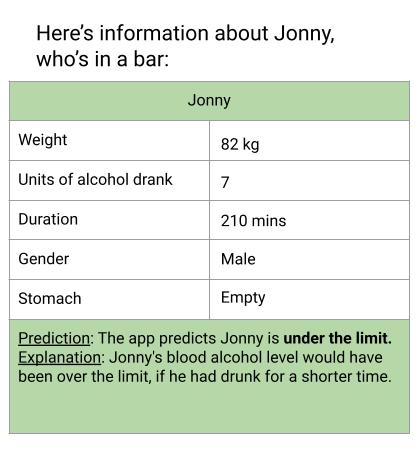 |
| --- | --- |
| 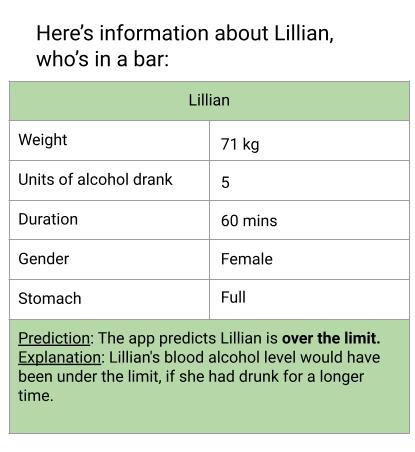 | 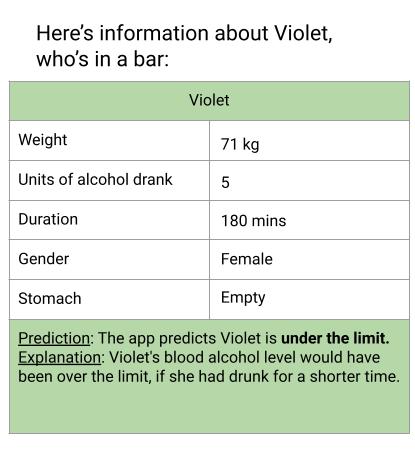 |
| 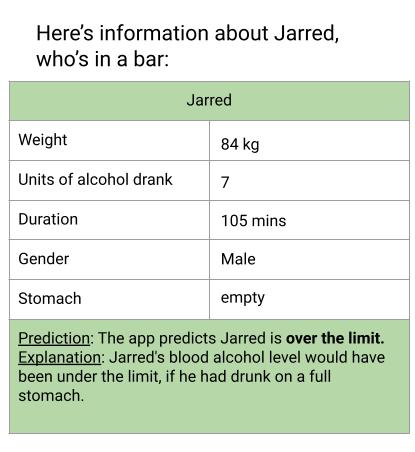 | 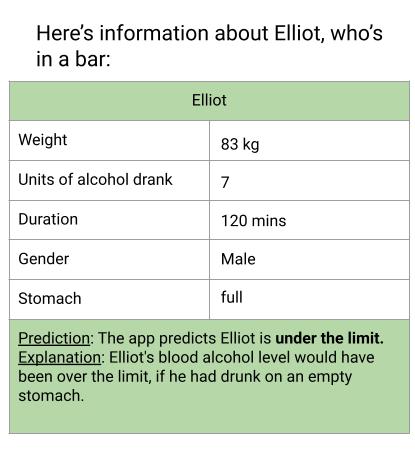 |
| 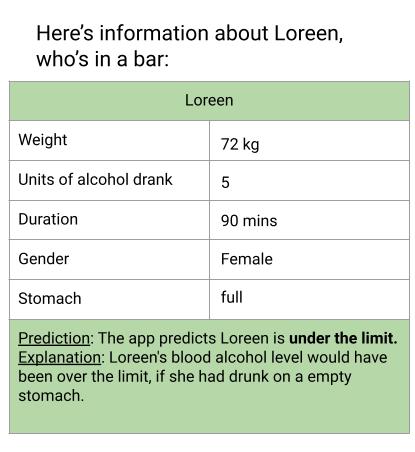 | 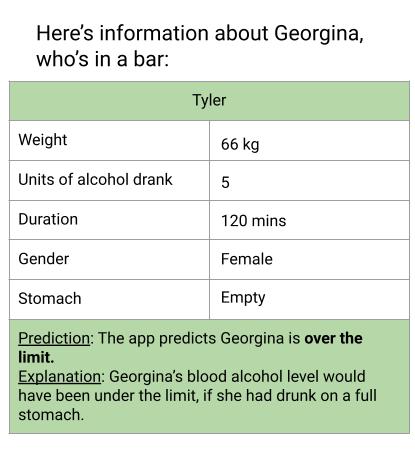 |
| 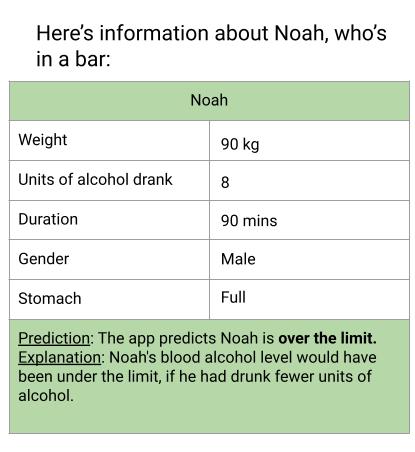 | 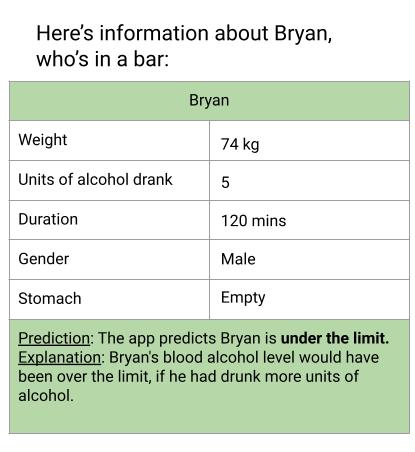 |
| 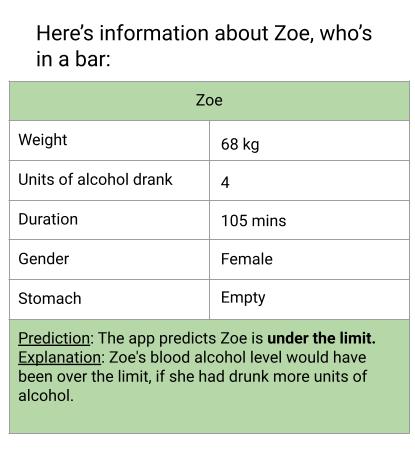 | 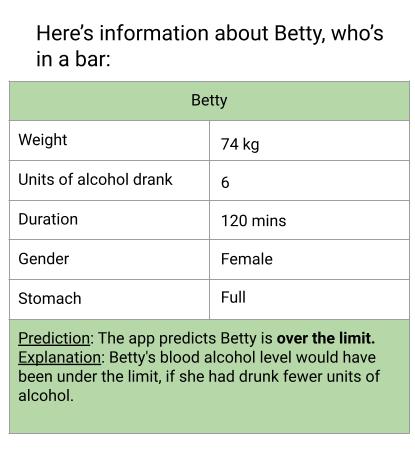 |
| 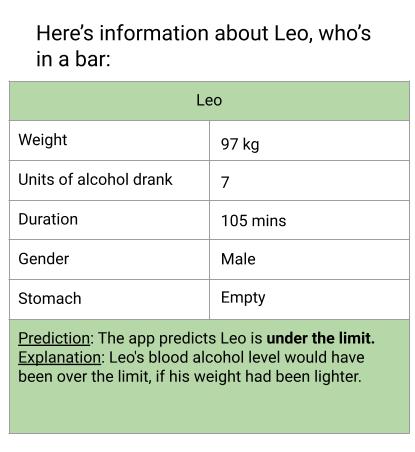 | 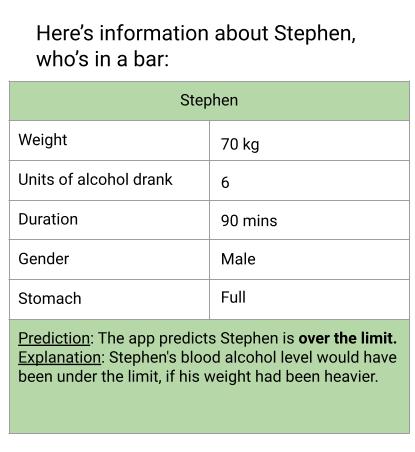 |
| 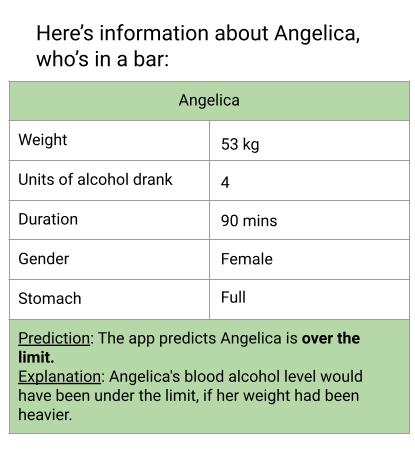 | 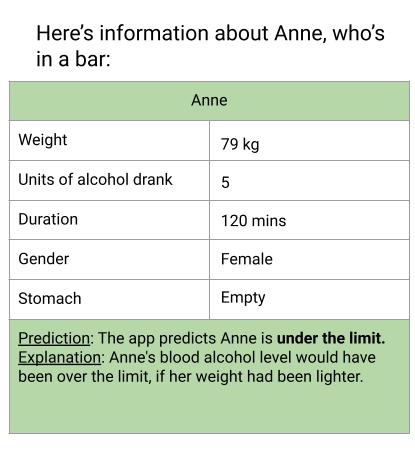 |
| **Attention Check** 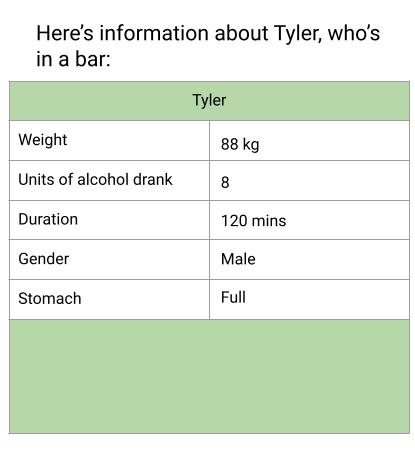 | |

***(b) The same 16 cases with causal explanations:***

| 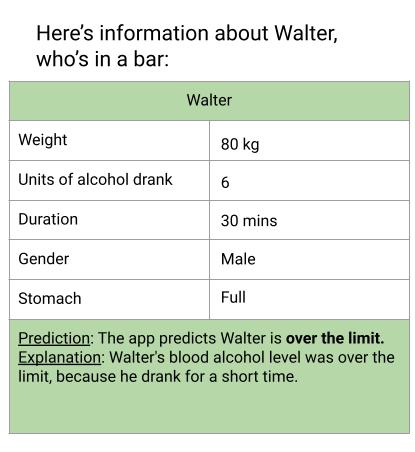 | 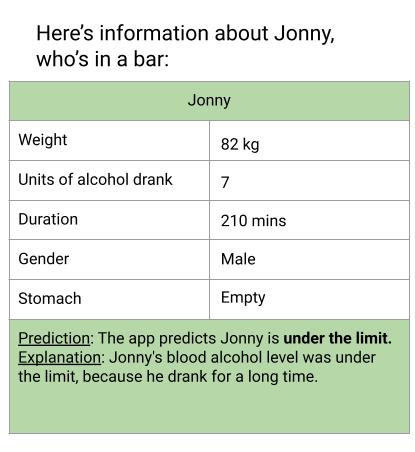 |
| --- | --- |
| 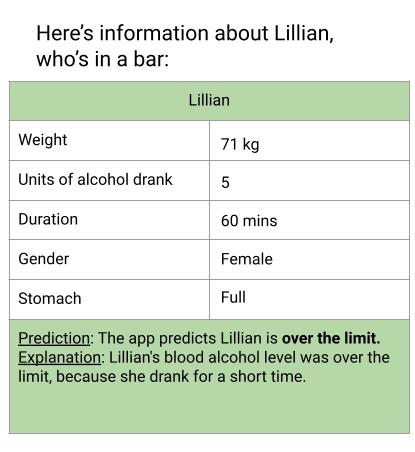 | 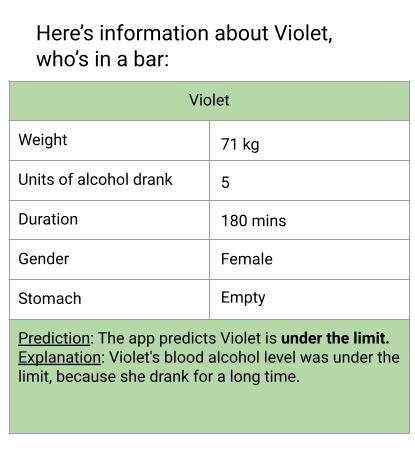 |
| 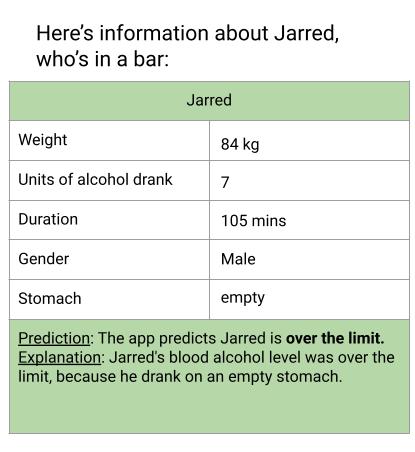 | 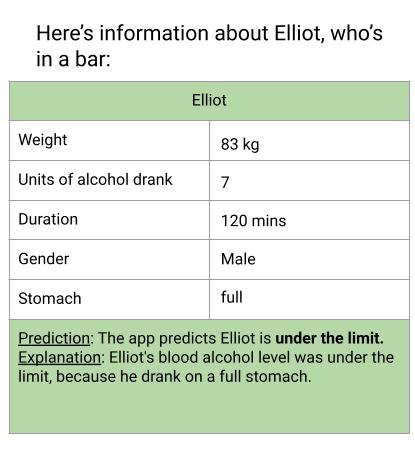 |
| 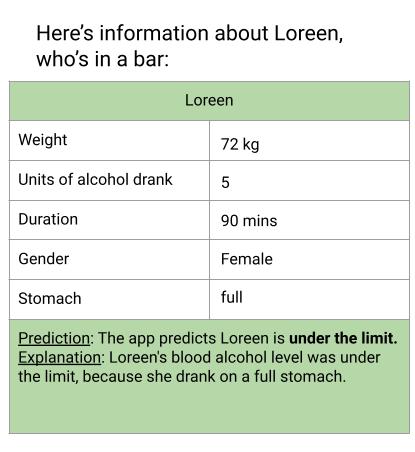 | 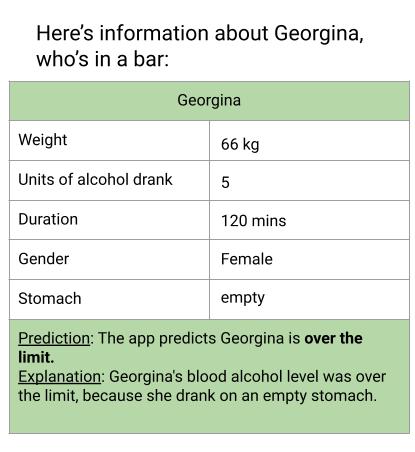 |
| 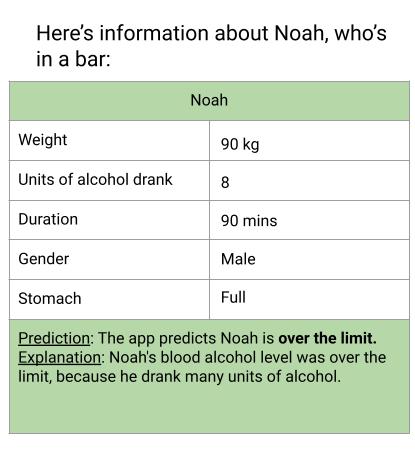 | 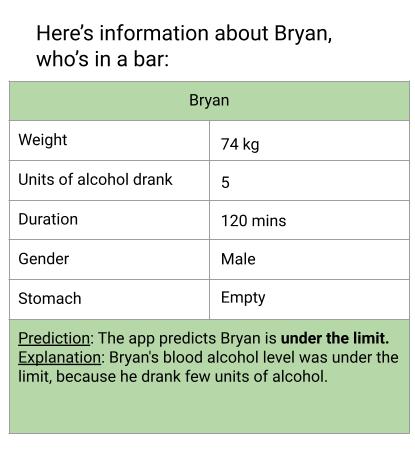 |
| 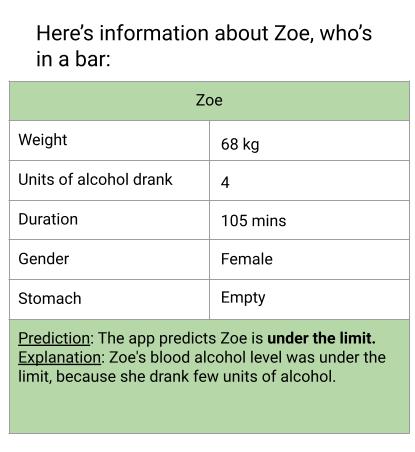 | 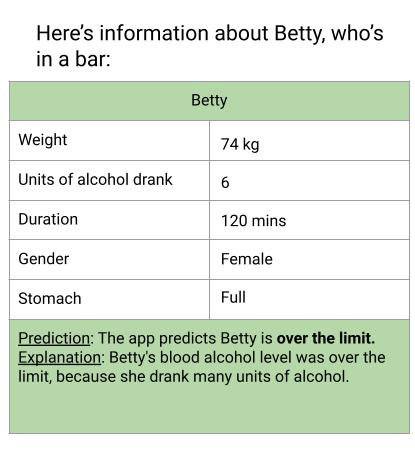 |
| 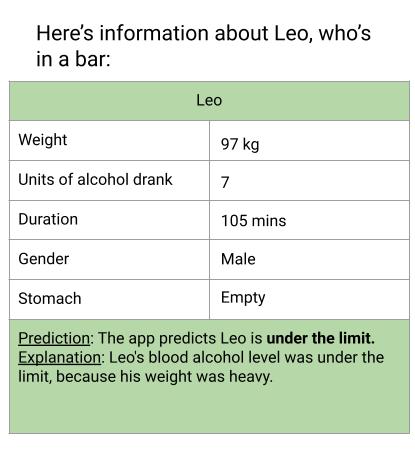 | 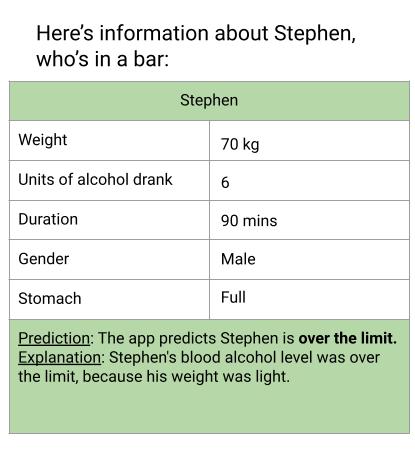 |
| 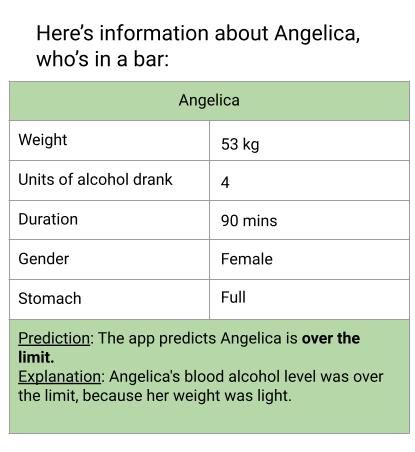 | 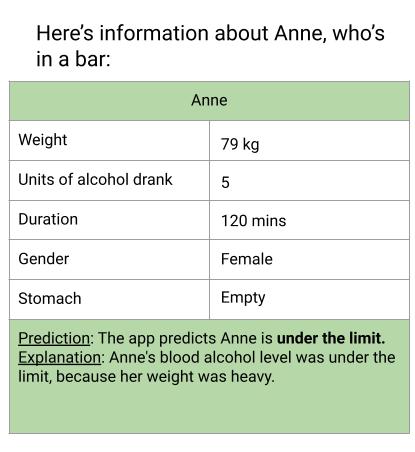 |
| **Attention Check**  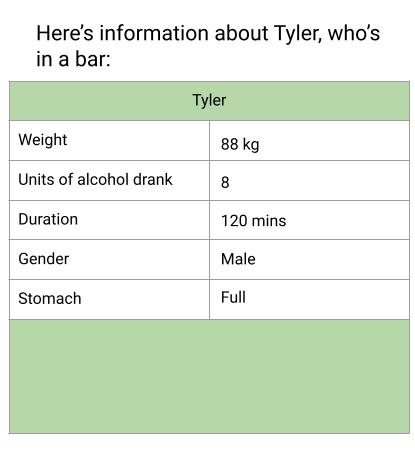 | |

***(c) The different set of 16 cases in the second part of the prediction experiments:***

| 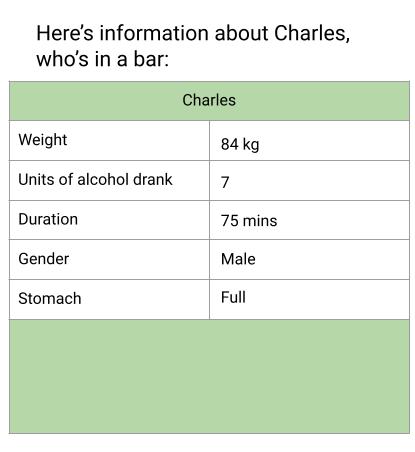 | 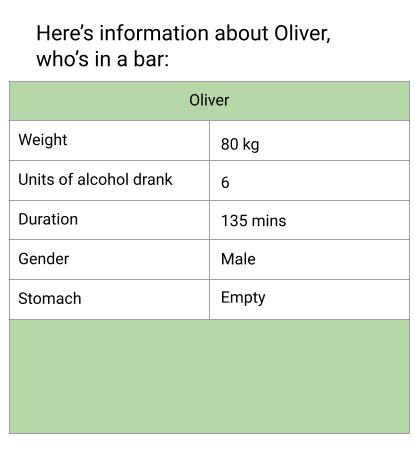 |
| --- | --- |
| 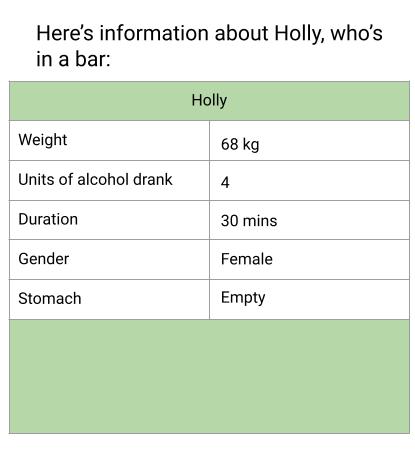 | 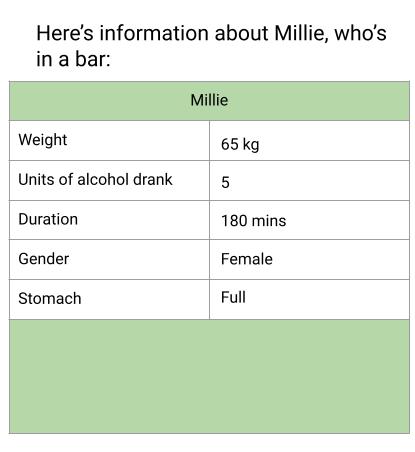 |
| 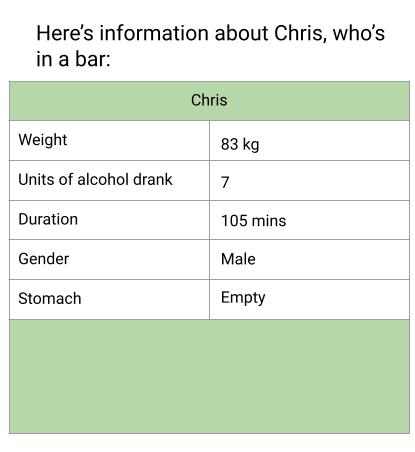 | 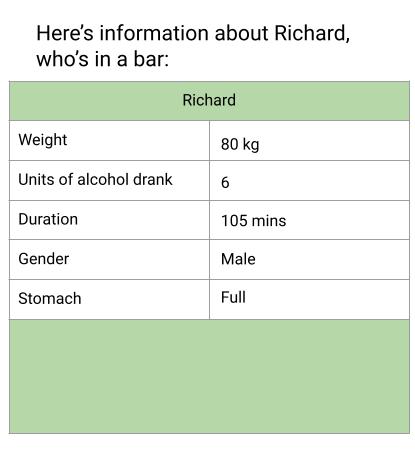 |
| 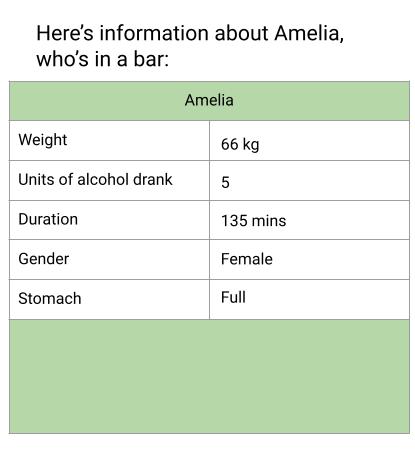 | 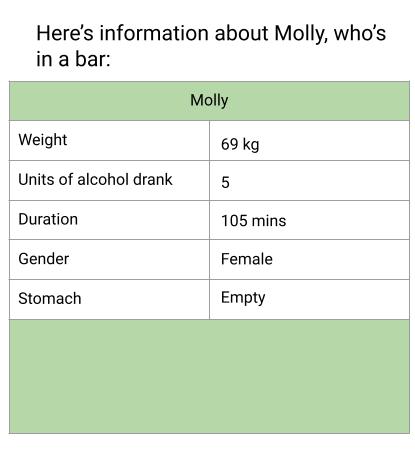 |
| 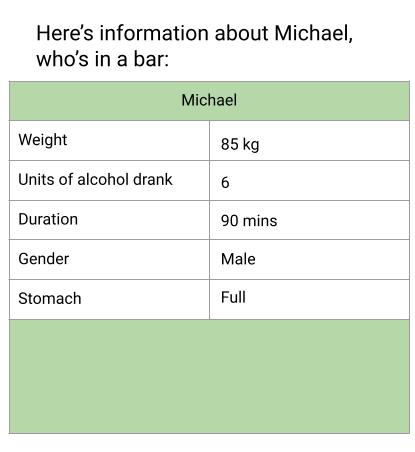 | 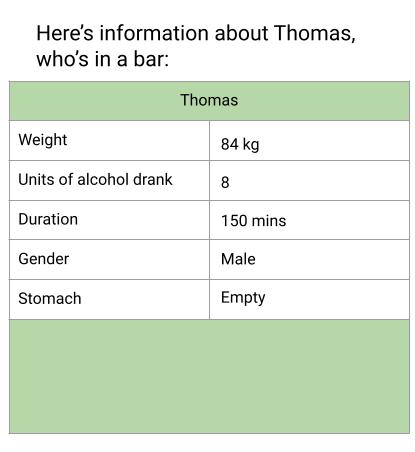 |
| 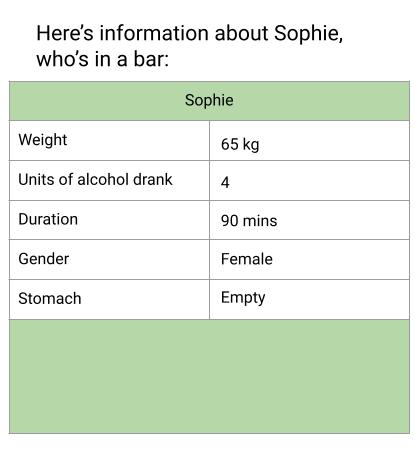 | 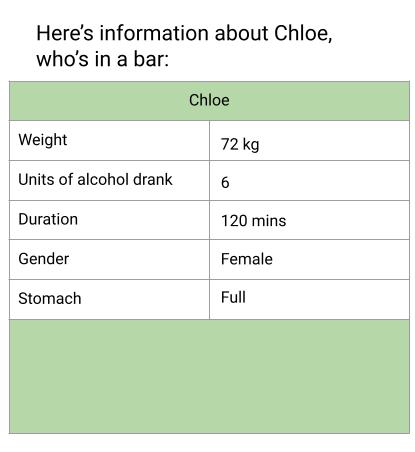 |
| 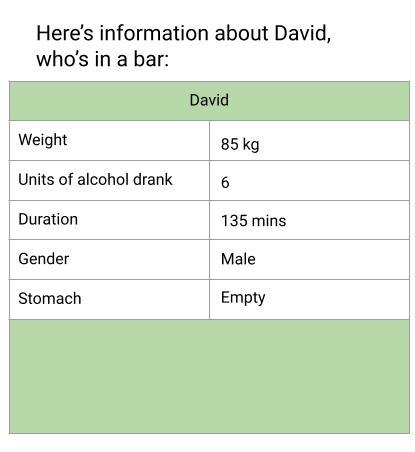 | 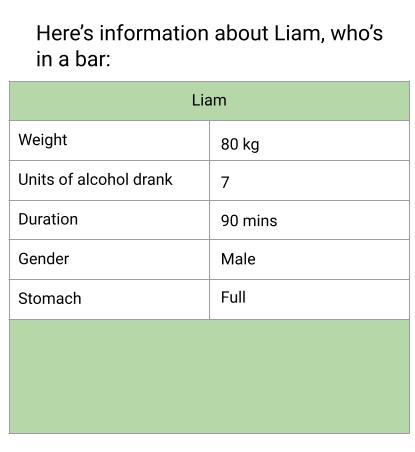 |
| 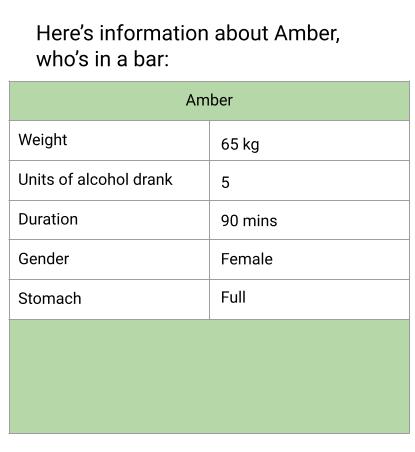 | 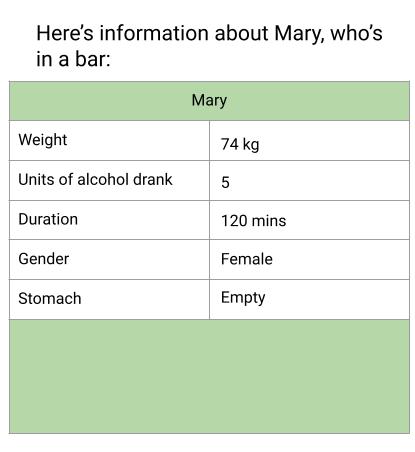 |
| **Attention Check** 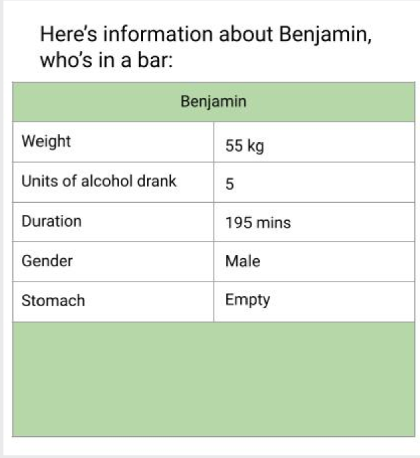 | |

***(d) An example of one of these 16 cases as used in the second part of the decision* *experiments:***


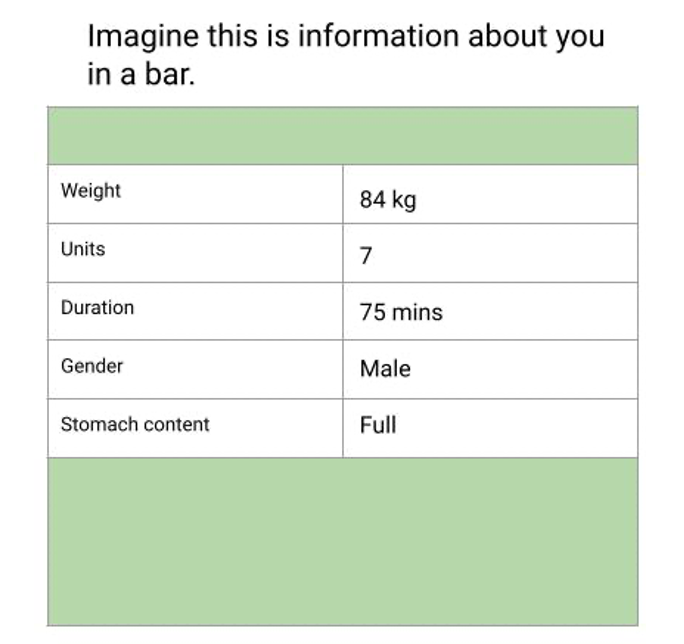


***Selection of cases from the AI case-base.***

Cases were selected from the AI case base generated by randomly perturbing five features: gender (male/female), weight (kg), duration of drinking (minutes at 15 minute intervals), stomach fullness (empty/full), and units of alcohol (see Warren, Smyth, & Keane, 2022). As noted in the paper, selection from the blood alcohol content (BAC) Artificial Intelligence case base was limited to cases with BAC proximal to the decision boundary (0.06 < BAC < 0.09; n = 10,163 cases). To ensure a meaningful difference between the query case and the counterfactual case, cases with BAC between 0.076 and 0.084 were excluded from being query cases, resulting in a total of n=7,463 cases. Out of the remaining pool of cases, 16 cases were randomly selected to be used in the first part of the experiment. To reduce the influence different features might have on participants’ perception of system predictions, each case was randomly assigned to one of the four feature groups, resulting in a total of 4 cases in the weight category, 4 cases in units of alcohol category, 4 cases in the duration category, and 4 cases in the stomach fullness category. Gender was represented equally with 50% of cases having the feature gender “Male”, and 50% “Female”. Each case then had the corresponding feature perturbed until the BAC outcome crossed the decision boundary, creating a counterfactual case. For example, a case assigned to the “units of alcohol” group had the feature “units of alcohol” perturbed, while the other three features (weight, duration, stomach fullness) remained static. Continuous variables were incremented in steps of 5 kg for weight, 1 unit for alcohol, and 15 minutes for duration, and the binary variable stomach fullness was assigned the inverse value. If the query case could not be perturbed to cross the decision boundary, a different case was randomly selected, and the same process was applied. If the perturbation was successful, the query case was selected to be included as a material in the experiment, while its counterfactual case was used as the basis for the explanation shown in the first part of the experiment. The 16 cases that were used in the second part of the experiment, were randomly selected from the pool of 7,463 cases, with an additional exclusion of the 16 cases that had already been selected as materials in the first part of the experiment, and the 16 cases that were used as the basis for the explanation. Two more cases with the same exclusions were randomly selected and they served as attention checks.

A baseline was also established for each of the variables. Each explanation focused on a specific feature, and all other features remained within their baseline range, e.g., the explanation in the paper in Figure 2 focused on units of alcohol, so weight and duration remained within their baseline range. Baseline weight range was 85±5 kg for males and 70±5 kg for females and was determined based on the available data for average weight in the countries from which participants were recruited. Weight below the baseline range was referred to in the explanations as “light” and weight above the baseline range was referred to as “heavy”. Baseline units of alcohol was based on the UK National Health Service’s Alcohol Advice in which binge drinking is classified as 8 units of alcohol in a single session for men and 6 for women. Units of 8 or more for men, and 6 or more for women were referred to in the explanations as “many”, and fewer than that were referred to as “few”. Baseline duration was based on the average duration one regular drink (pint of beer / glass of wine) stays in the system. The baseline range for duration was set at 120±30 minutes. Duration below the baseline range was referred to in the explanations as “short”, and above the baseline range was referred to as “long”.

**Materials for the unfamiliar domain (ChemSafe).** We provide (a) the set of 16 cases for the first part of the experiment with counterfactual explanations, (b) the same cases with causal explanations, (c) the different set of 16 cases for the prediction part, (d) an example used in the decision experiments.

***(a) The set of 16 cases with counterfactual explanations:***

| 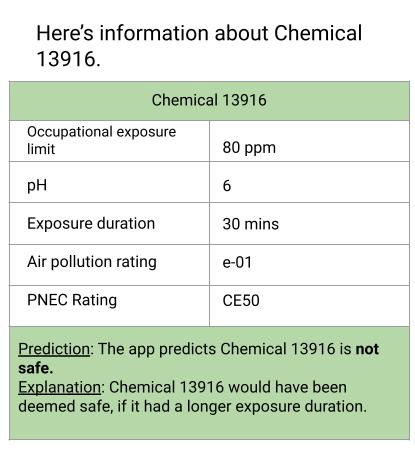 | 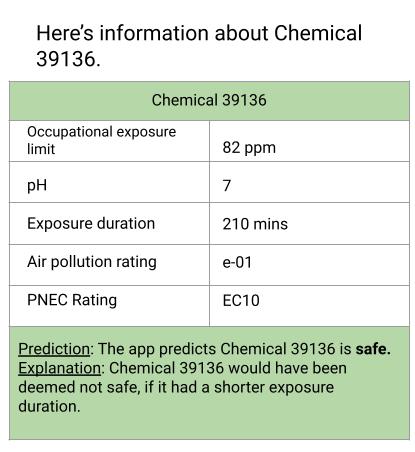 |
| --- | --- |
| 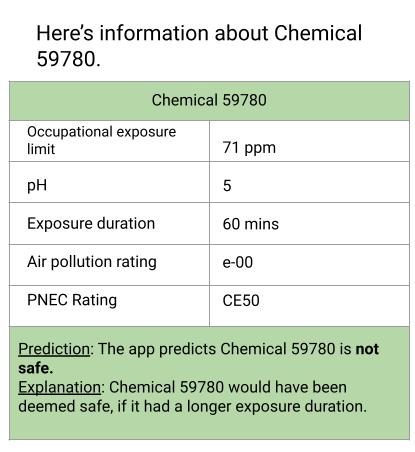 | 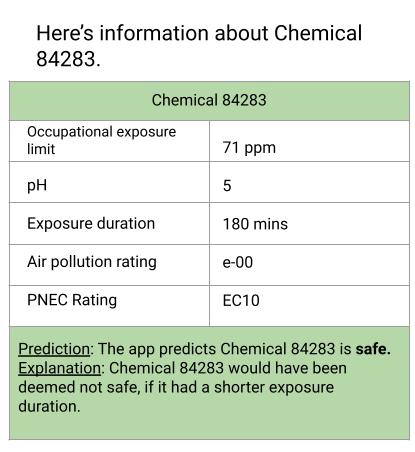 |
| 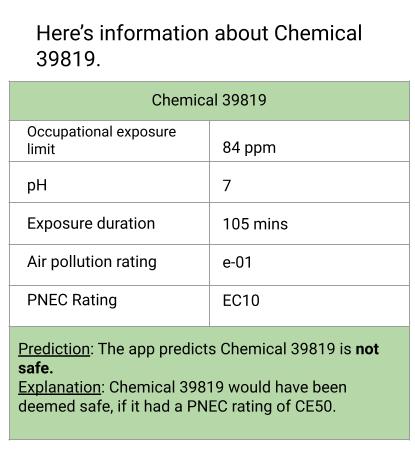 | 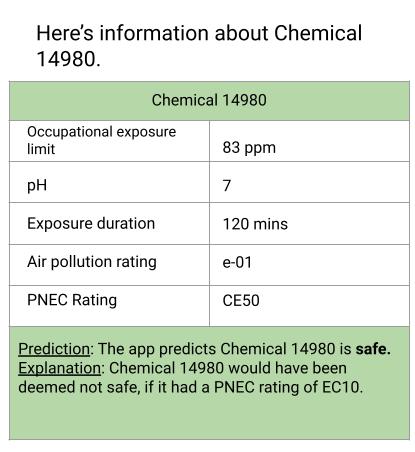 |
| 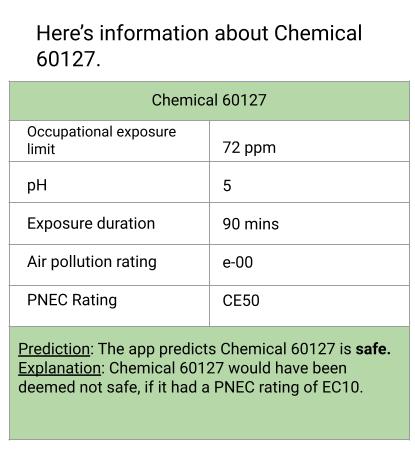 | 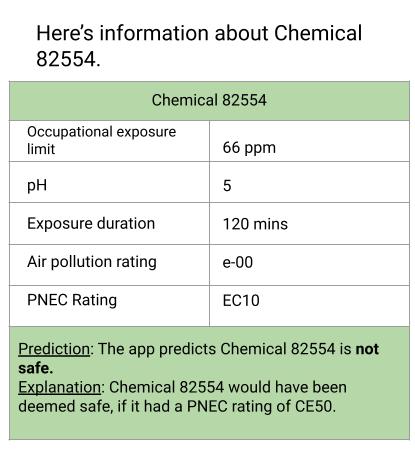 |
| 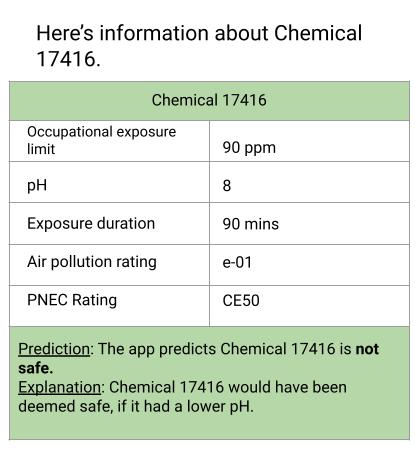 | 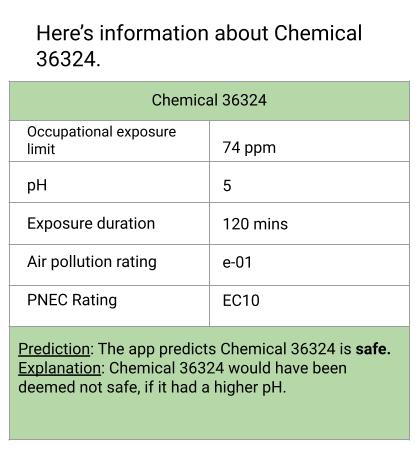 |
| 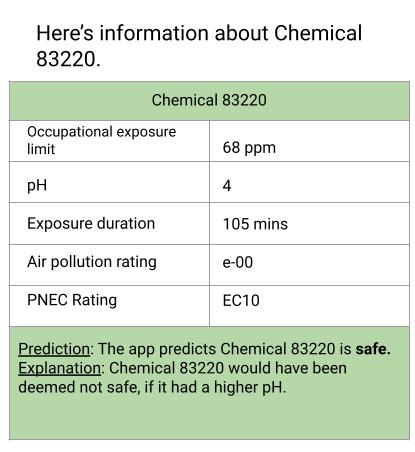 | 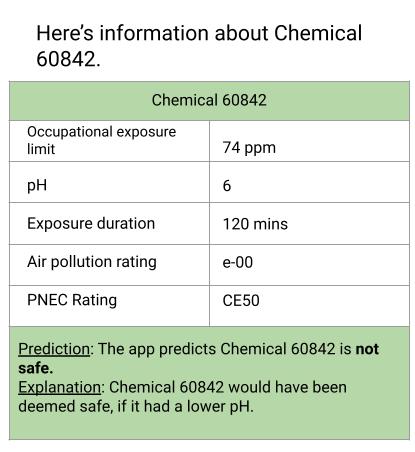 |
| 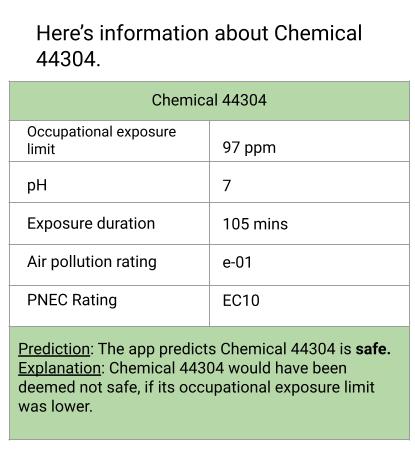 | 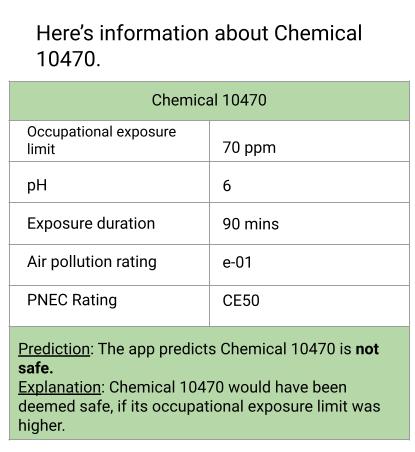 |
| 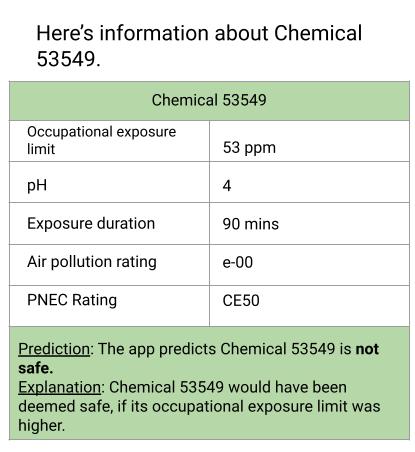 | 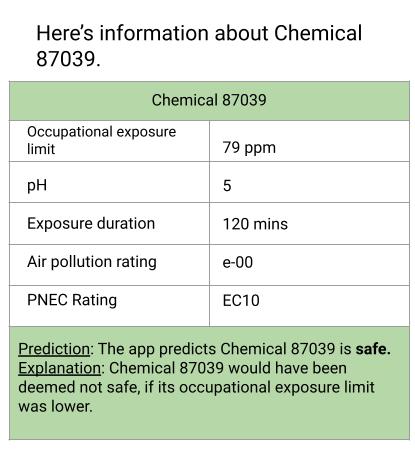 |
| **Attention Check** 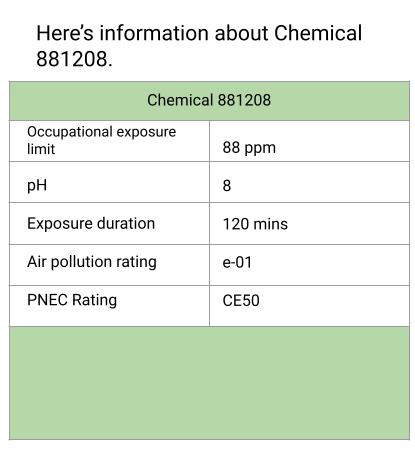 | |

***(b) The same set of 16 cases with causal explanations:***

| 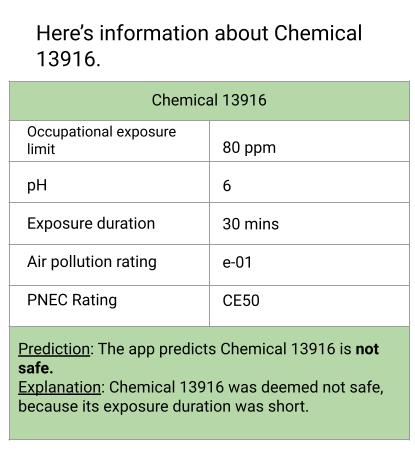 | 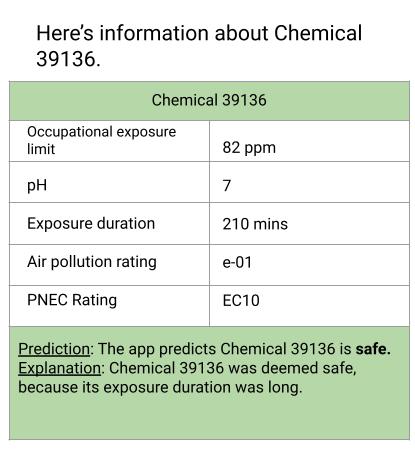 |
| --- | --- |
| 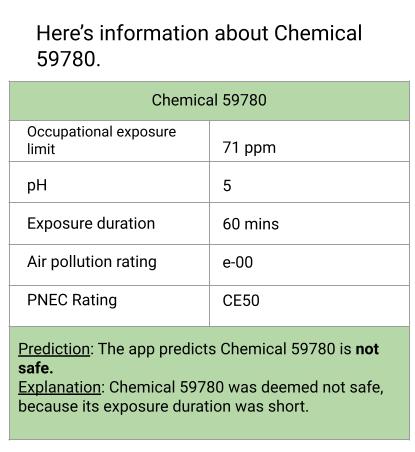 | 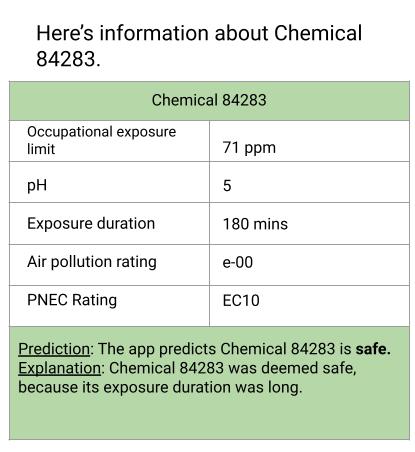 |
| 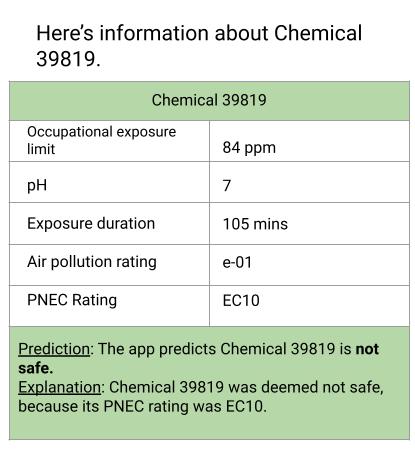 | 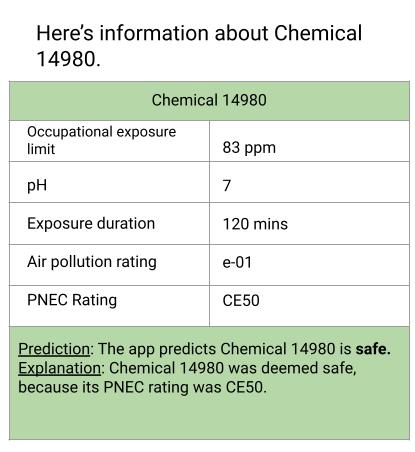 |
| 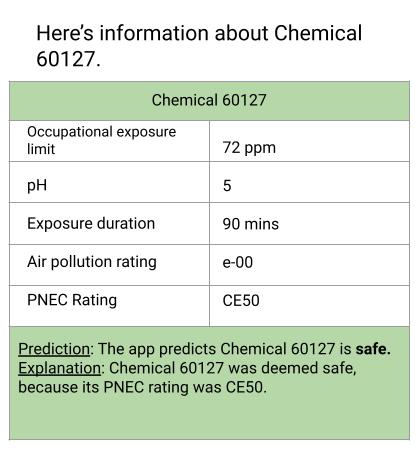 | 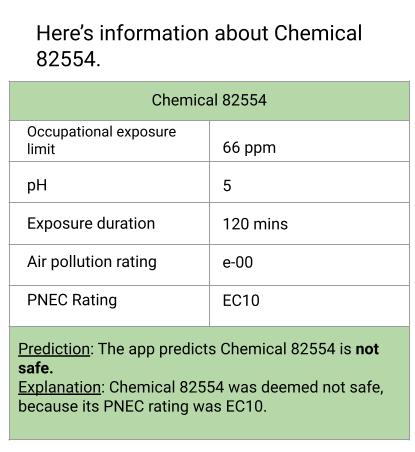 |
| 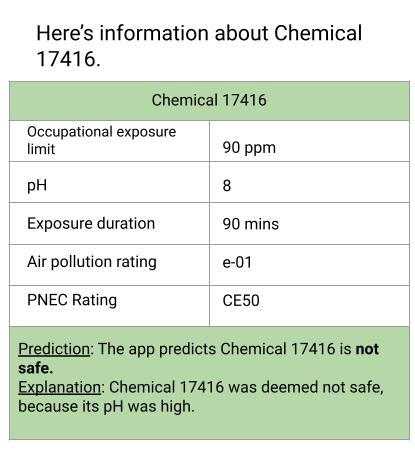 | 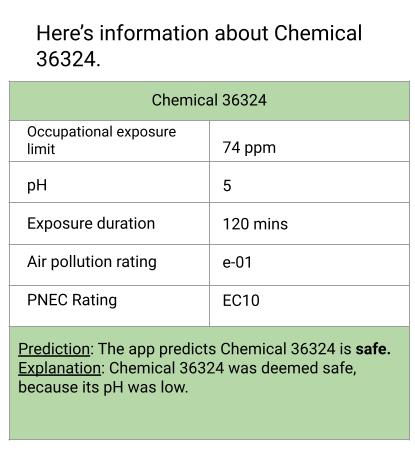 |
| 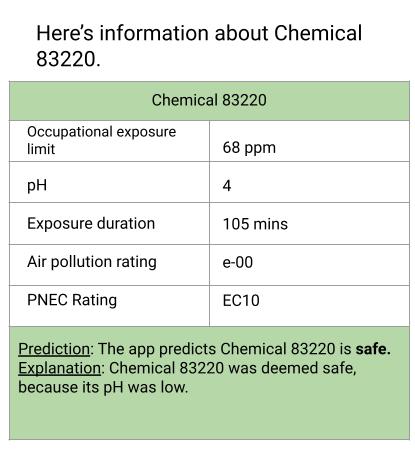 | 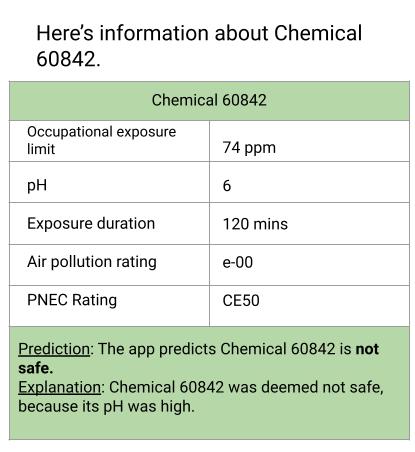 |
| 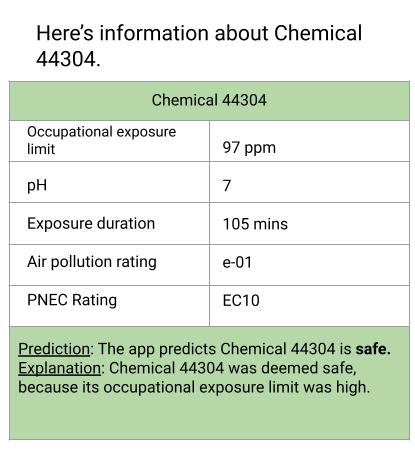 | 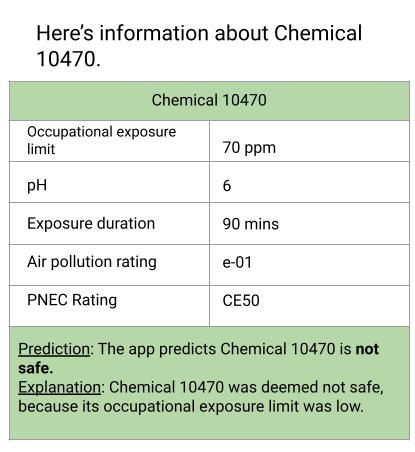 |
| 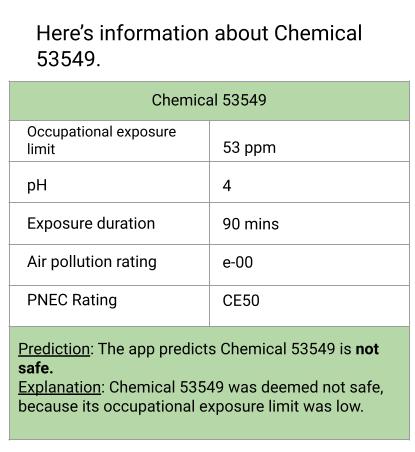 | 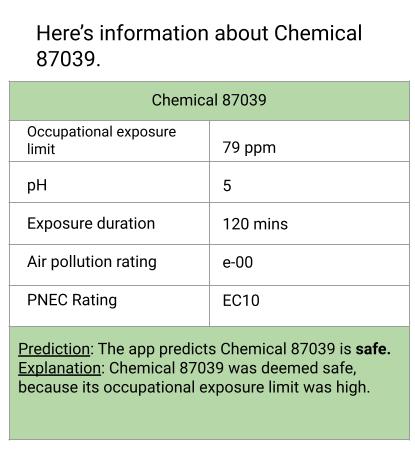 |
| **Attention Check** 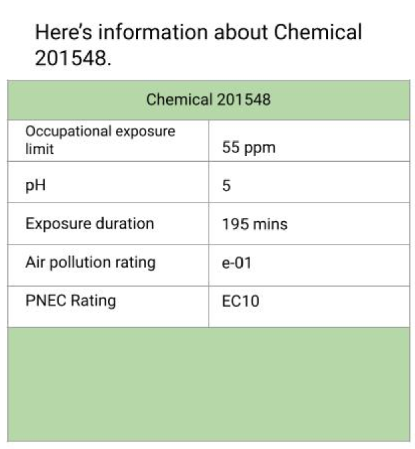 | |

***(c) The different set of 16 cases for the prediction part of the experiments:***

| 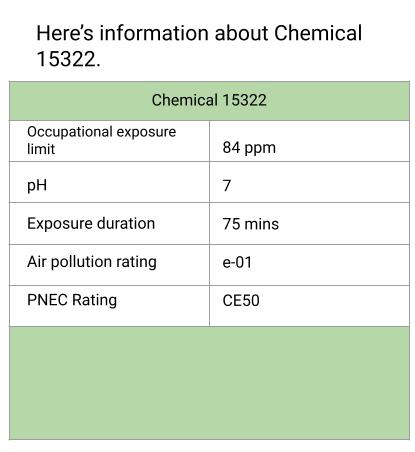 | 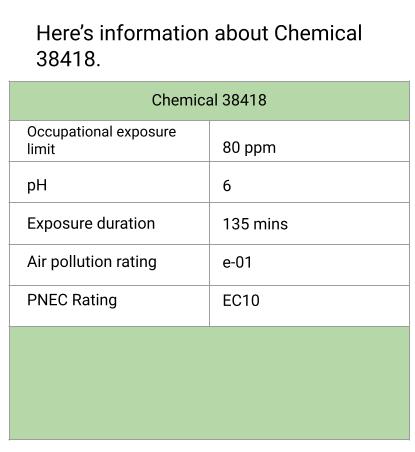 |
| --- | --- |
| 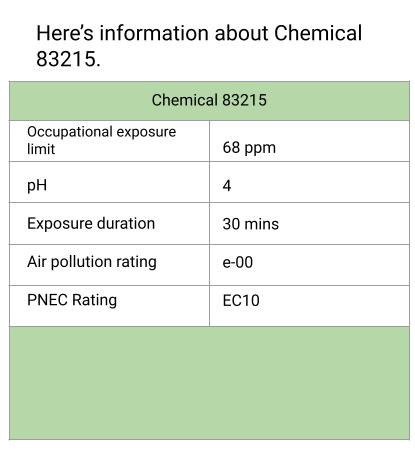 | 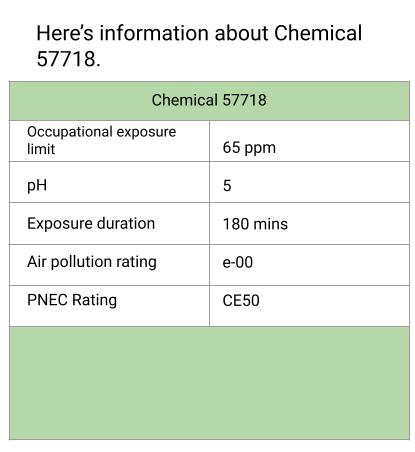 |
| 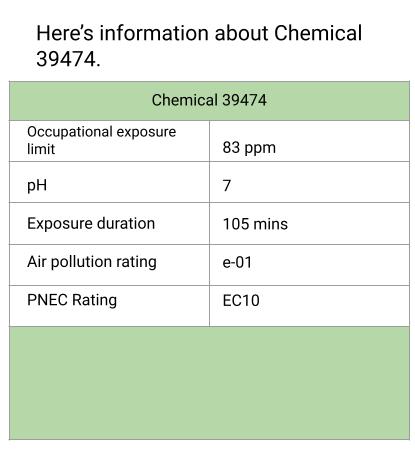 | 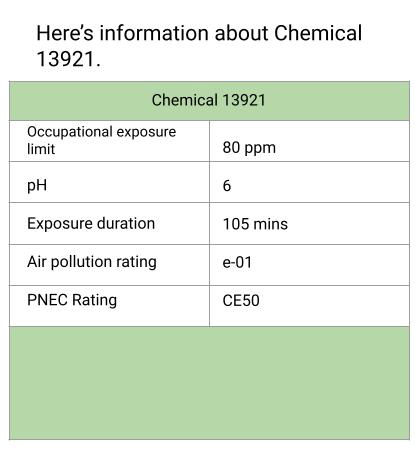 |
| 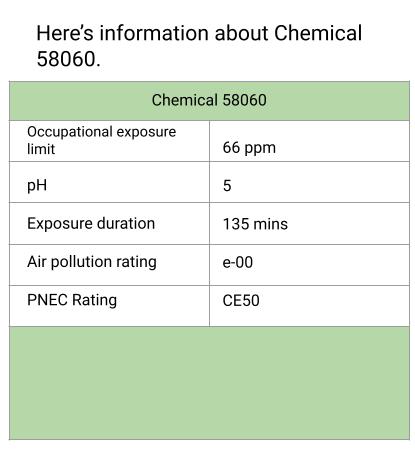 | 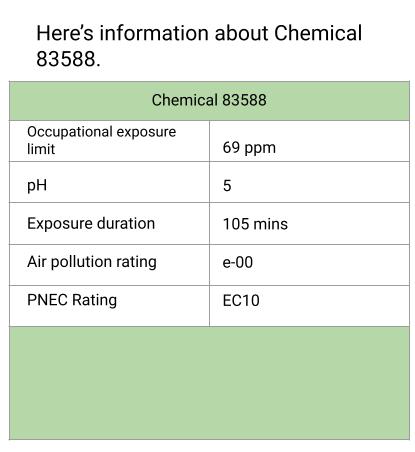 |
| 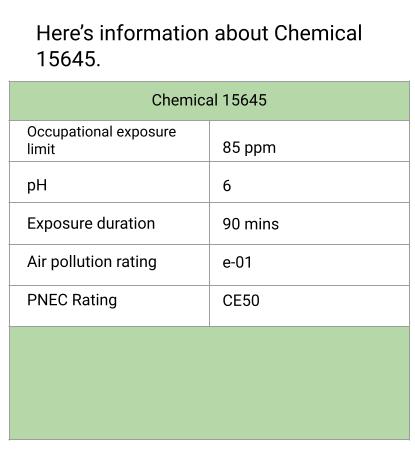 | 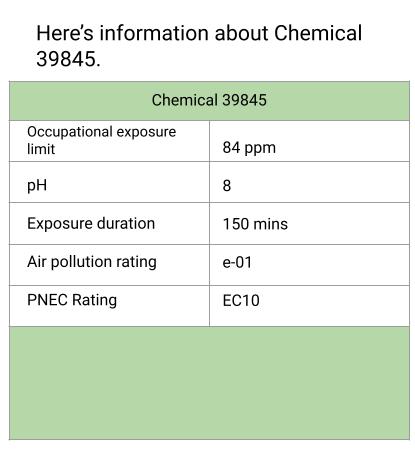 |
| 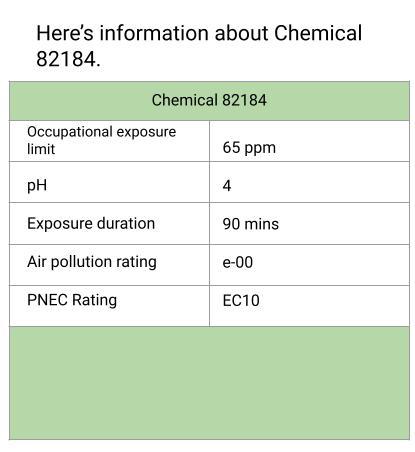 | 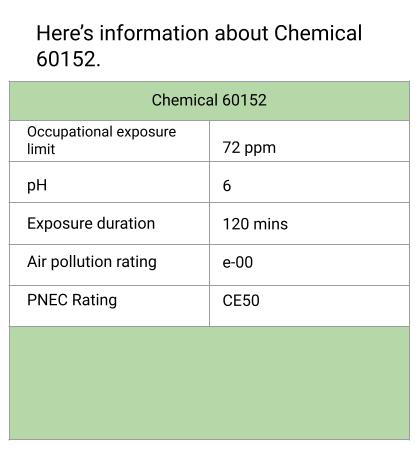 |
| 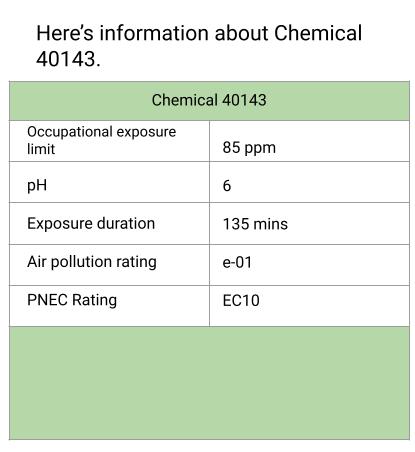 | 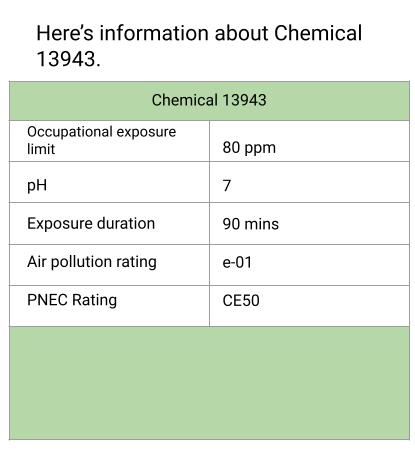 |
|  |  |
| **Attention Check** | |

***(d) An example of one used in the decision experiments:***

**Conversion of SafeLimit to ChemSafe*.*** As noted in the paper, the cases for the *ChemSafe* app were created to be analogous to the blood alcohol cases used in *SafeLimit* and the same cases were used from the *SafeLimit* with modifications to reduce familiarity, i.e., features and units from the BAC system were converted to chemical safety technical terms, while the values and case logic remained the same, as outlined in Table S1.

**Table S1.** Feature conversion chart for cases in the familiar and unfamiliar domains.

| **Familiar (SafeLimit App)** | | **Unfamiliar (ChemSafe App)** | |
| --- | --- | --- | --- |
| ***Feature*** | **Unit** | ***Feature*** | **Unit** |
| *Weight* | kg | *Occupational exposure limit* | ppm |
| *Units drank* | Units | *pH* | Units |
| *Duration* | min (minutes) | *Exposure duration* | min (minutes) |
| *Gender* | Male | *Air pollution rating* | e-01 |
|  | Female |  | e-00 |
| *Stomach fullness* | Empty | *PNEC rating* | EC10 |
|  | Full |  | CE50 |

**Additional statistical analyses**

***Experiment 1***

Levene’s test was significant for the ***explanation helpfulness*** ***judgments*** indicating violation of the assumption of homogeneity of variance and so we followed the recommended procedure for unequal n’s (Gignac, 2019) of repeating the ANOVA using equal numbers in each group (reducing all groups to the smallest n, i.e., n=40, by random selection for each group via SPSS). The same results were found, i.e., explanation, F (1, 156) = 20.238, *p* < .001, η_p_^2^ = .12; familiarity, F (1, 156) = 13.756, *p* < .001, η_p_^2^ = .08; interaction, F (1.56) =.005, *p* =.95.

In Experiment 1, for the ***explanation helpfulness judgments***, the same direction of difference was observed for almost all of the items. Participants judged counterfactual explanations more helpful than causal ones for each one of the 16 items, and they judged explanations more helpful in the familiar domain than the unfamiliar one for 14 of the 16 items, binomial test, p < .0001. A further check of the consistency of items showed that explanations were judged more helpful for the 8 items with bad outcomes than the 8 items with good outcomes, F (1, 173) = 5.536, p < .02, η_p_^2^=0.031, as shown in a 2 (explanation) x 2 (familiarity) x 2 (outcome: bad, i.e., over the limit/unsafe, vs good, i.e., under the limit/safe) ANOVA with repeated measures on the last factor. Nonetheless, counterfactual explanations were judged more helpful than causal ones for both sorts of outcomes, bad: t (175) = 3.294, p < .001, CI [-.5626, -.14106], and good: t (175) = 5.037, p < .001, CI [-.73486, -.3211], in the decomposition of the interaction of outcome with explanation, F (1, 173) = 5.553, p < .02, η_p_^2^=0.031, and explanations were judged more helpful in the familiar domain than the unfamiliar one, for both sorts of outcomes, bad: t (175) = 4.116, p < .001, CI [-.6408, -.2255], and good: t (175) = 2.465, p < .015, CI [-.4900, -.0542], in the decomposition of the interaction of outcome with familiarity, F (1, 173) = 5.045, p < .026, η_p_^2^=0.028 (and the three variables did not interact together).

For ***prediction accuracy***, the same direction of difference was also observed for most of the items. Participants made as many correct predictions whether they had been given counterfactual or causal explanations for 10 of the 16 items (less than 5% of a difference), binomial p < .01; they made more correct predictions (greater than 5%) when they had been given counterfactual rather than causal explanations for 3 items, and more when they had been given causal rather than counterfactual explanations for 3 items. They made more correct predictions for the familiar domain than the unfamiliar one, for 12 of the 16 items, binomial test, p < .0006. The same pattern occurred for good and bad outcomes, as shown by no effect of outcome, F (1, 173) = .976, p = .325, in the 2 (explanation) x 2 (familiarity) x 2 (outcome: bad vs good) ANOVA, and no interaction of outcome with familiarity, F (1, 173) = .215, p = .644, or explanation, F (1, 173) = 1.815, p = .18 (and the three variables did not interact).

Levene’s test was significant for the ***confidence judgments*** in Experiment 1 and so once again we followed the recommended procedure for unequal n’s (Gignac, 2019). The same results were found when we repeated the ANOVA reducing all groups to the smallest n, i.e., explanation, F (1, 156) = .884, *p* = .35; familiarity, F (1, 156) = 8.048, *p* < .001, η_p_^2^ = .05; interaction, F (1.56) =.00, *p* =.99.

***Experiment 2***

For the **explanation helpfulness judgments**, the same direction of difference was observed for most of the items. Participants judged counterfactual explanations more helpful than causal ones for 14 of the 16 items, binomial test, p < .0001, and they judged explanations more helpful in the familiar domain than the unfamiliar one for 11 of the 16 items, binomial p < .003. A further check of the consistency of items showed that explanations were judged more helpful for items with bad outcomes than good ones, F (1, 169) = 12.573, p < .001, η_p_^2^=0.069*,* in a 2 (explanation) x 2 (familiarity) x 2 (outcome: good vs bad) ANOVA. Nonetheless, counterfactual explanations were judged somewhat more helpful than causal ones for both sorts of outcomes, as shown by no interaction of outcome with explanation, F (1, 169) = 1.212, p < .273; explanations were judged more helpful in the familiar domain than the unfamiliar one for bad outcomes, t (171) = 4.561, p < .001, CI [-.68399, -.27076], but there was no difference for good outcomes, t (171) = 0.155, p < .877, in the decomposition of the interaction of outcome with familiarity, F (1, 169) = 26.475, p < .0001, η_p_^2^=0.135 (and the three variables did not interact together).

For **decision accuracy** the same direction of difference between counterfactual and causal explanations was not observed for all items and we report the analysis in the paper. As reported there, participants’ decisions were more accurate when they had been given counterfactual rather than causal explanations (more than 5% difference) for 8 of the 16 items, but more accurate when they had been given causal rather than counterfactual explanations for 6 items, and there was no difference (greater than 5%) for 2 items. In testing the consistency of items further, participants’ decisions were more accurate for the 8 items with bad outcomes than the 8 items with good outcomes, F (1, 169) = 48.923, p < .0001, η_p_^2^=.224, in the 2 (explanation) x 2 (familiarity) x 2 (outcome: good -under the limit/safe to handle, vs bad - over the limit/unsafe to handle) ANOVA with repeated measures on the third factor. Their decisions about items with good outcomes were more accurate when they had received counterfactual explanations rather than causal ones, t (171) = 3.482, p < .001, CI [-.23773, -.0657], but more accurate about items with bad outcomes when they had received causal explanations rather than counterfactual ones, t (171) = 2.196, p < .029, CI [.00822, .15413], in the decomposition of the interaction of outcome with explanation, F (1, 169) = 10.594, p < .001, η_p_^2^=.059.

The same direction of difference between familiar and unfamiliar domains was observed for most items. Participants were more accurate in the familiar domain than the unfamiliar one for 10 of the 16 items, binomial test, p <.01. They were more accurate in the familiar domain than the unfamiliar one for bad outcomes, t (171) =5.156, p < .0001, CI [-.24851, -.11091], but there was no difference for good outcomes, t (171) = 1.637, p = .103, in the decomposition of the interaction of outcome with familiarity, F (1, 169) = 12.615, p < .0001, η_p_^2^=.069 (and the three variables did not interact together).

***Experiment 3a and 3b***

Levene’s test was significant for the confidence measure in Experiment 3a and so once again we followed the recommended procedure for unequal n’s (Gignac, 2019). The same results were found when we repeated the ANOVA (reducing all groups to the smallest n, i.e., n=43 by random selection via SPSS), i.e., familiarity, F (1, 168) = 9.527, *p* = .001, η_p_^2^ =.08; explanation type, F (1, 168) = .295, *p* = .59; interaction, F (1, 168) =.613, p=.44.

**(iii). Materials for the incorrect decisions for the familiar domain (SafeLimit).** We provide (a) the set of 16 cases for the first part of the experiments, with counterfactual explanations, (b) the same cases with causal explanations. Note the different set of 16 cases for the prediction part or decision part are identical to those in (c) and (d) in 1 above.

***(a) The 16 cases with counterfactual explanations:***

|  |  |
| --- | --- |
|  |  |
|  |  |
|  |  |
|  |  |
|  |  |
|  |  |
|  |  |
| **Attention Check** | |

***(b) The same 16 cases with causal explanations:***

|  |  |
| --- | --- |
|  |  |
|  |  |
|  |  |
|  |  |
|  |  |
|  |  |
|  |  |
| **Attention Check** | |

**(iv). Materials for the incorrect decisions of the unfamiliar domain (ChemSafe).** We provide (a) the set of 16 cases for the first part of the experiments, with counterfactual explanations, (b) the same cases, with causal explanations. Note the different set of 16 cases for the prediction or decision part are identical to those in (c) and (d) in 2 above.

***(a) The 16 cases with counterfactual explanations:***

|  |  |
| --- | --- |
|  |  |
|  |  |
|  |  |
|  |  |
|  |  |
|  |  |
|  |  |
| **Attention Check** | |

***(b) The same 16 cases with causal explanations:***

|  |  |
| --- | --- |
|  |  |
|  |  |
|  |  |
|  |  |
|  |  |
|  |  |
|  |  |
| **Attention Check** | |

**Explanation satisfaction scale (Hoffman, et al., 2018).**

1. From the explanation, I understand how the app works.

2. This explanation of how the app works is satisfying.

3. This explanation of how the app works has sufficient detail.

4. This explanation of how the app works seems complete.

5. This explanation of how the app works tells me how to use it.

6. This explanation of how the app works is useful to my goals.

7. This explanation of the app shows me how accurate the app is.

8. This explanation lets me judge when I should trust and not trust the app.

**Trust scale (Hoffman et al., 2018).**

1. I am confident in the app. I feel that it works well.

2. The outputs of the app are very predictable.

3. The tool is very reliable. I can count on it to be correct all the time.

4. I feel safe that when I rely on the app I will get the right answers.

5. The app is efficient in that it works very quickly.

6. I am wary of the app *(reverse scored)*

7. The app can perform the task better than a novice human user.

8. I like using the system for decision making.

For each question, participants indicated their answer on the following scale:

| 5 | 4 | 3 | 2 | 1 |
| --- | --- | --- | --- | --- |
| I agree strongly | I agree somewhat | I’m neutral about it | I disagree somewhat | I disagree strongly |

**Memory Check Questions**

*Familiar Condition:* “In this study, you were shown predictions about people being over or under the legal alcohol limit to drive, made from 5 different features about them. Can you recall what these 5 features were? Select all that you remember.” The following items were presented one under the other with tick boxes beside each one:

*Body Mass Index (BMI); Duration of drinking period; Alcohol tolerance; Height; Gender; Age; Units of alcohol consumed; Stomach fullness; Weight; Alcohol strength.*

*Unfamiliar Condition:* “In this study, you were shown predictions about chemicals being either safe or not safe, made from 5 different features about them. Can you recall what these 5 features were? Select all that you remember.” The following items were presented one under the other with tick boxes beside each one:

*PNEC rating; Chemical Index; Chemical Age; Chemical tolerance; Air pollution rating; Occupational exposure limit; Exposure duration; Chemical Barrier Height; pH; Strength.*

**Analyses of Explanation Satisfaction and Trust scales**

In each of the experiments, participants completed the DARPA Explanation Satisfaction and Trust scales (Hoffman et al., 2018) after completing the two main parts of the experiment.

***Experiment 1****.* Participants in Experiment 1 were equally satisfied with counterfactual explanations and causal ones, F (1, 173) = .03, p = .88; and more satisfied with explanations given for the familiar domain than the unfamiliar one, F (1, 173) = 15.10, *p* < .001, η_p_^2^ = .11; and the two variables interacted, F(1, 173) = 4.41, *p* = .04, η_p_^2^ = .03. To decompose the interaction, a series of paired comparisons using independent sample t-tests was carried out, with a Bonferroni correction for four comparisons of *p* =.0125. For the familiar domain, participants given counterfactual explanations were as satisfied as those given causal ones, t (92) = -1.61, *p* = .11; similarly, for the unfamiliar domain, participants given counterfactual explanations were as satisfied as those given causal ones, t(82) = 1.39, *p* = .17. The interaction instead arises mainly because given counterfactual explanations, participants were more satisfied in the familiar domain than the unfamiliar one, t(87) = 5.02, *p* < .001, Cohen’s d = .80; and given causal explanations, they were equally satisfied in the familiar and unfamiliar domain, t (86) = 1.74, *p* = .09. The results are consistent with the local explanation helpfulness judgments for familiarity although not for explanations.

For Trust ratings, participants rated the AI system as trustworthy equally given counterfactual explanations as causal ones, F (1, 177) = .45, *p* = .50, and equally for the familiar domain as the unfamiliar one, F (1, 177) = .88, *p* = .35; the two variables did not interact, F (1, 177) = 1.08, *p* = .30.

***Experiment 2.*** Participants in Experiment 2 were somewhat more satisfied with counterfactual explanations than causal ones, F(1, 173) = 3.23, *p* = .07, η_p_^2^ = .02, and more satisfied with explanations given for the familiar domain than the unfamiliar one, F(1, 173) = 14.71, *p* < .001, η_p_^2^ = .12, and the two variables did not interact, F(1, 173) = .00, *p* = 1.00. The results are consistent with the local explanation helpfulness judgments for familiarity and explanations.

For Trust ratings, participants rated the AI system as trustworthy more given counterfactual explanations as causal ones, F(1, 173) = 3.96, *p* = .048, η_p_^2^ = .02, and equally for the familiar domain as the unfamiliar one, F(1, 173) = .32, *p* = .35, the two variables did not interact, F(1, 173) = .32, *p* = .57.

***Experiments 3a and 3b.*** Participants were equally satisfied with explanations given for the familiar domain as the unfamiliar one, in Experiment 3a, F (1, 180) = 1.63, *p* = .20; and Experiment 3b, F(1, 186) = .11, *p* = .75; they were equally satisfied with counterfactual explanations and causal ones, in Experiment 3a, F (1, 180) = .95, *p* = .33, and more satisfied with counterfactual explanations than causal ones in Experiment 3b, F(1, 186) = 14.71, *p* = .02, η_p_^2^ = .03; the two variables did not interact, in Experiment 3a, F (1, 180) = .05, *p* = .82, or Experiment 3b, F(1, 186) = 1.48, *p* = .23. The results are consistent with the local explanation helpfulness judgments for explanations although not for familiarity.

For Trust ratings, participants rated the AI system as less trustworthy given the familiar domain than the unfamiliar domain in Experiment 3a, F(1, 184) = 24.64, p < .001, η_p_^2^ = .12, and Experiment 3b, F(1, 186) = 26.25, p < .001, η_p_^2^ = .13; they rated it as trustworthy equally given counterfactual explanations as causal ones in Experiment 3a, F (1, 184) = .29, p = .59; and more trustworthy given counterfactual explanations than causal one in Experiment 3b, F(1, 186) = 7.37, p = .007, η_p_^2^ = .04; the two variables did not interact, in Experiment 3a, F (1, 184) = .01, p = .91, or Experiment 3b, F(1, 186) = 1.27, p = .26. Table S2 provides a summary of the results of the DARPA scales for the four experiments, and includes also the results for the other dependant variables as presented in Table 1 in the paper.

**Table S2** A summary of the results of the four experiments, including the DARPA scales

|  | Experiment 1 | Experiment 2 | Experiment 3a | Experiment 3b |
| --- | --- | --- | --- | --- |
| *Participants’ Judgments:* | Predictions | Decisions | Predictions | Decisions |
| *AI system outputs:* | Correct | Correct | Incorrect | Incorrect |
| Explanation Helpfulness | Familiar | Familiar | Familiar | Familiar |
|  | > | > | < | < |
|  | Unfamiliar | Unfamiliar | Unfamiliar | Unfamiliar |
|  | Counterfactual | Counterfactual | Counterfactual | Counterfactual |
|  | > | ≥ * | = | = |
|  | Causal | Causal | Causal | Causal |
| Accuracy | Familiar | Familiar | Familiar | Familiar |
|  | > | > | **<** | < |
|  | Unfamiliar | Unfamiliar | Unfamiliar | Unfamiliar |
|  | Counterfactual | Counterfactual | Counterfactual | Counterfactual |
|  | = | > | = | = |
|  | Causal | Causal | Causal | Causal |
| Confidence | Familiar | Familiar | Familiar | Familiar |
|  | > | > | > | > |
|  | Unfamiliar | Unfamiliar | Unfamiliar | Unfamiliar |
|  | Counterfactual | Counterfactual | Counterfactual | Counterfactual |
|  | = | = | = | = |
|  | Causal | Causal | Causal | Causal |
| Explainability | Familiar | Familiar | Familiar | Familiar |
|  | > | > | = | = |
|  | Unfamiliar | Unfamiliar | Unfamiliar | Unfamiliar |
|  | Counterfactual | Counterfactual | Counterfactual | Counterfactual |
|  | = | ≥ * | = | > |
|  | Causal^+^ | Causal | Causal | Causal |
| Trust | Familiar | Familiar | Familiar | Familiar |
|  | = | = | < | < |
|  | Unfamiliar | Unfamiliar | Unfamiliar | Unfamiliar |
|  | Counterfactual | Counterfactual | Counterfactual | Counterfactual |
|  | = | > | = | > |
|  | Causal | Causal | Causal | Causal |

≥ * p = .068

+ Interaction of familiarity and explanation type: see text.

**Questions assessing participants’ beliefs against ever drinking and driving, or handling potentially unsafe chemicals.**

*Familiar domain*

I believe that people who have had even one drink should not drink and drive.

I believe that it is acceptable for people who are under the legal limit to drive.

*Unfamiliar domain*

I believe you should never handle chemicals you are unfamiliar with.

I believe that it is acceptable to handle chemicals that are deemed safe.

For each question, participants indicated their answer on the following scale:

| 5 | 4 | 3 | 2 | 1 |
| --- | --- | --- | --- | --- |
| I agree strongly | I agree somewhat | I’m neutral about it | I disagree somewhat | I disagree strongly |

References:

Gignac, G. E. (2019). How2statsbook (Online Edition 1). Perth, Australia. http://www.how2statsbook.com/p/chapters.html
